# Towards an automated analysis of bacterial peptidoglycan structure

**DOI:** 10.1007/s00216-016-9857-5

**Published:** 2016-08-13

**Authors:** Marshall Bern, Richard Beniston, Stéphane Mesnage

**Affiliations:** 1 Protein Metrics Inc., 1622 San Carlos Ave., Suite C, San Carlos, CA 94070 USA; 2 Biological Mass Spectrometry Facility biOMICS, University of Sheffield, Brook Hill Road, Sheffield, S3 7HF UK; 3 Department of Molecular Biology and Biotechnology, University of Sheffield, Western Bank, Sheffield, S10 2TN UK; 4 Covance Laboratories Limited, Otley Road, Harrogate, North Yorkshire HG3 1PY UK

**Keywords:** Peptidoglycan, Cell wall, Muropeptides, Proteomics, Tandem mass spectrometry, Glycoproteomics, Cross-link

## Abstract

Peptidoglycan (PG) is an essential component of the bacterial cell envelope. This macromolecule consists of glycan chains alternating *N*-acetylglucosamine and *N*-acetylmuramic acid, cross-linked by short peptides containing nonstandard amino acids. Structural analysis of PG usually involves enzymatic digestion of glycan strands and separation of disaccharide peptides by reversed-phase HPLC followed by collection of individual peaks for MALDI-TOF and/or tandem mass spectrometry. Here, we report a novel strategy using shotgun proteomics techniques for a systematic and unbiased structural analysis of PG using high-resolution mass spectrometry and automated analysis of HCD and ETD fragmentation spectra with the Byonic software. Using the PG of the nosocomial pathogen *Clostridium difficile* as a proof of concept, we show that this high-throughput approach allows the identification of all PG monomers and dimers previously described, leaving only disambiguation of 3–3 and 4–3 cross-linking as a manual step. Our analysis confirms previous findings that *C. difficile* peptidoglycans include mainly deacetylated *N*-acetylglucosamine residues and 3–3 cross-links. The analysis also revealed a number of low abundance muropeptides with peptide sequences not previously reported.

**Electronic supplementary material:**

The online version of this article (doi:10.1007/s00216-016-9857-5) contains supplementary material, which is available to authorized users.

**Graphical Abstract:**
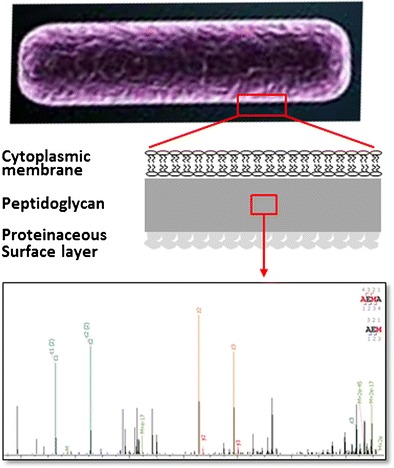
The bacterial cell envelope includes plasma membrane, peptidoglycan, and surface layer. Peptidoglycan is unique to bacteria and the target of the most important antibiotics; here it is analyzed by mass spectrometry.

## Introduction

The major and essential polymer within the bacterial cell envelope is peptidoglycan (PG), which forms a single bag-shaped macromolecule (or sacculus) around the cell [1]. PG is unique to the bacterial kingdom, and its synthesis is the target of the most clinically important antibiotics ever discovered such as the beta-lactams (penicillin) and glycopeptides (vancomycin). In addition to a role as an exoskeleton that confers cell shape and resistance to the intracellular osmotic pressure, PG is used as a scaffold for the display of a myriad of polymers and proteins at the cell surface.

**Fig. 1:**
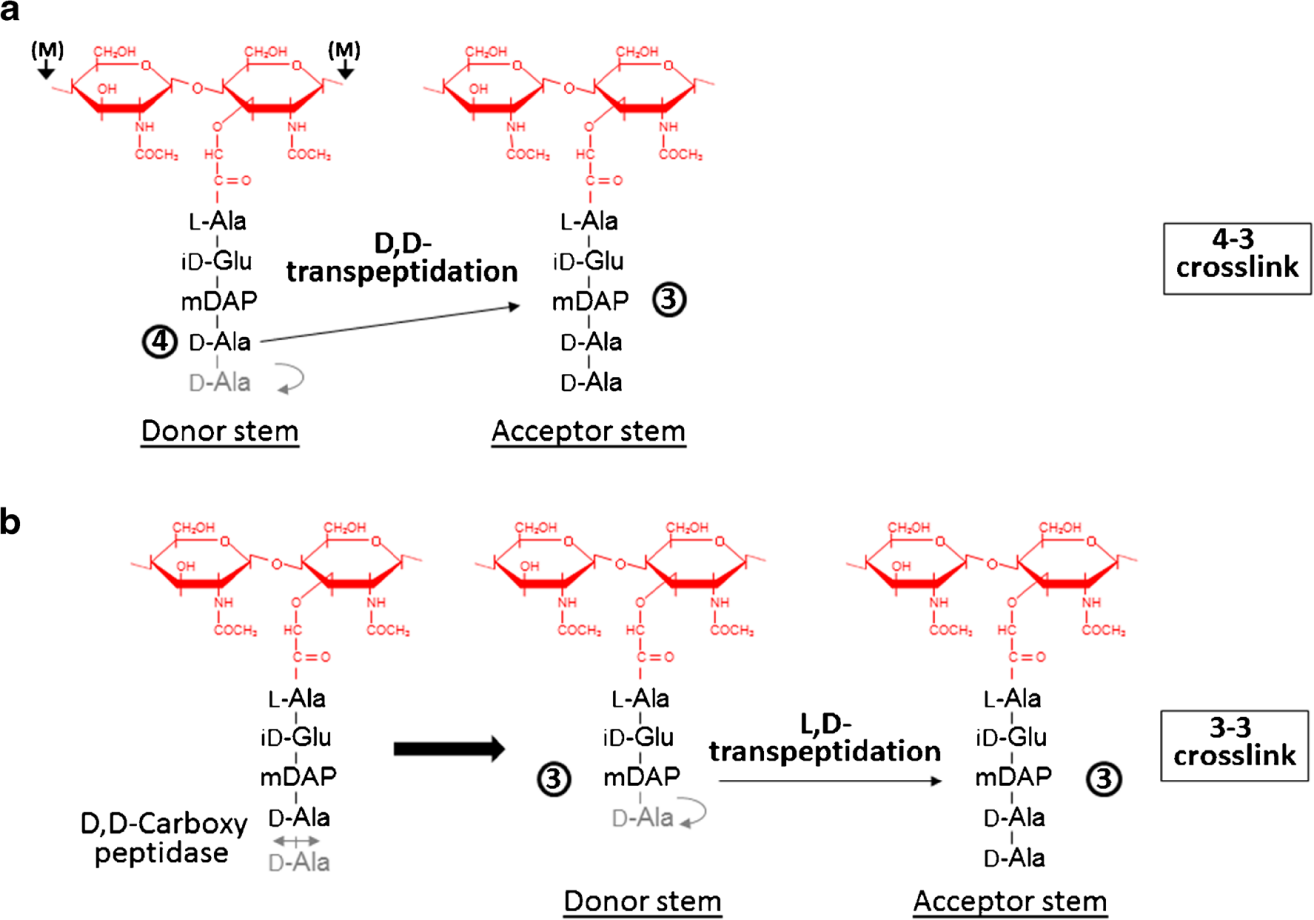
Composition and polymerization of bacterial peptidoglycan (*PG*). PG building blocks, cut from larger PG chains with mutanolysin cleavages indicated by (*M*), correspond to the disaccharide-pentapeptide GlcN-MurNAc-l-Ala-iD-Glu-mDAP-d-Ala-d-Ala. PG components can be cross-linked by two distinct mechanisms: (**a**) d,d-transpeptidation results in the formation of a 4–3 bond between a donor and an acceptor stem; (**b**) l,d-transpeptidation follows cleavage of d-Ala in position 5 and results in the formation of a 3–3 bond between a donor and an acceptor stem

PG composition is variable amongst the bacteria, but is usually highly conserved within a given species [2]. The macromolecule is made of beta-1,4-linked glycan chains alternating *N*-acetylglucosamine (GlcNAc) and *N*-acetylmuramic acid (MurNAc). MurNAc residues are substituted via a lactyl group by pentapeptide stems most frequently made of l-Ala-iso-d-Glu-*m*eso-DAP (or l-Lys)-d-Ala-d-Ala (mDAP, *meso*-diaminopimelic acid). During growth, PG precursors are assembled in the cytoplasm and translocated at the cell surface where they are polymerized. Whilst glycan chain polymerization is conserved, two types of enzymes can cross-link peptide stems (Fig. 1). d,d-Transpeptidases recognize the C-terminal d-Ala-d-Ala extremity of a donor stem. They form a covalent intermediate with the d-Ala in position 4 and link the COOH group of this residue to the NH_2_ group of the mDAP residue in position 3 of an acceptor peptide stem, thereby forming 4–3 cross-links. d,d-Transpeptidases are also called penicillin-binding proteins (PBPs) as they are inhibited irreversibly by beta-lactam antibiotics, which are d-Ala-d-Ala structural analogs. Peptidoglycan cross-links can also result from the activity of l,d-transpeptidases. These enzymes recognize the last two residues of a tetrapeptide donor stem substrate generated by a carboxypeptidase. They form a covalent intermediate with the meso-DAP in position 3 and link the COOH group of this residue to the NH_2_ group of the mDAP of an acceptor peptide stem. l,d-Transpeptidases form 3–3 cross-links and are not inhibited by beta-lactam antibiotics. In addition to distinct modes of polymerization, several enzymatic modifications take place during growth, a process called “PG remodeling.” Such modifications can occur on glycan strands (e.g., *O*-acetylation or de-*N*-acetylation), reviewed by Vollmer [3], or on peptide stems (e.g., amidation of γd-Glu, cleavage of C-terminal residues, or amino acid substitutions mediated by l,d-transpeptidases), reviewed by Vollmer et al. [1].

Surprisingly, the strategy proposed nearly 30 years ago by Bernd Glauner [4] is still used routinely to explore PG structure. It involves the purification of PG sacculi, followed by digestion with lysozyme to generate disaccharide peptides (muropeptides) that are separated by reversed-phase HPLC. Individual fractions are collected, desalted and analysed by MS and MS/MS. Improvement of chromatography equipment has increased the throughput of PG analyses, allowing the separation of several micrograms of muropeptides in less than 30 min [5, 6]. Recent studies have reported PG structural analysis using online LC-ESI-MS [7] or LC-ESI-MS/MS [8–10] for the identification of muropeptides. Although these approaches overcome a major limitation (time-consuming offline fractionation), they still rely on manual analysis of the major ions and, hence, have limited capability to search for new PG components, likely biasing the analysis towards previously identified species. To the best of our knowledge, no automated analysis of peptidoglycan tandem mass spectra has been reported before.

Here, we describe a novel strategy for a systematic, unbiased, structural analysis of PG that combines high mass accuracy and automated analysis of HCD and ETD fragmentation spectra using Byonic and Byologic software (Protein Metrics, San Carlos, CA). Using the PG of the nosocomial pathogen *Clostridium difficile* as a proof of concept, we show that this systematic, unbiased, “bottom up” approach allows the identification and quantitation of all PG monomers and dimers previously described, leaving only disambiguation of 3–3 and 4–3 cross-linking as a manual step. Our analysis confirms previous findings [11] that *C. difficile* peptidoglycans include mainly deacetylated GlcNAc residues (GlcN) and 3–3 cross-links, and that acceptor peptides often include glycine substituted for the d-Ala in position 4. Byonic’s “wildcard” search, which allows any mass delta on any one residue, also finds a number of low abundance muropeptides with peptide sequences not previously reported. Variable residues are located at the C-terminus of acceptor peptides, after the mDAP in position 3.

## Experimental section

### Extraction and purification of peptidoglycan samples

Fifty milliliters of TY broth was inoculated with an overnight culture of *C. difficile* strain 630 [11] at a starting OD_600_ of 0.05. Exponentially growing cells (OD600 = 0.45) were collected by centrifugation at 4500 rpm for 10 min at 25 °C. The pellet was resuspended in 10 mL of boiling MilliQ water (MQ) before the addition of an equal volume of boiling SDS 8 %. After 30 min at 100 °C, the cells were left to cool down to room temperature. Insoluble cell walls were pelleted at 45,000*g* for 15 min and washed five times using 30 mL of MilliQ water. Proteins covalently bound to peptidoglycan were removed by pronase treatment (final concentration of 2 mg/mL for 4 h at 60 °C). Protease-treated cell walls were washed 6 times with 30 mL of MilliQ water before covalently bound polymers were removed by incubation in 1 M HCl for 5 h at 37 °C. Insoluble pure peptidoglycan was washed 6 times with MilliQ water, freeze-dried and resuspended at a final concentration of 10 mg/mL.

### Preparation of soluble muropeptides

One milligram of purified peptidoglycan (HCl-treated cell walls) were digested overnight in 50 mM phosphate buffer (pH 5.5) supplemented with 100 μg of mutanolysin in a final volume of 125 μL. Following heat inactivation of mutanolysin (5 min at 100 °C), soluble disaccharide peptides were then mixed with an equal volume of 250 mM borate buffer (pH 9.25) and reduced with 1 % (*m*/*v*) sodium borohydride. After 20 min at room temperature, the pH was adjusted to 4.5 using phosphoric acid. Reduced muropeptides were desalted by rp-HPLC using water–formic acid 0.1 % (*v*/*v*) as buffer A and one-step elution with 25 % acetonitrile (*v*/*v*)–formic acid 0.1 % (*v*/*v*) gradient.

#### Data acquisition

Samples were analysed online using an UltiMate 3000 RSLCnano LC System (Dionex) coupled to an LTQ Orbitrap Elite hybrid mass spectrometer (Thermo) equipped with a nanospray ion source. Desalted muropeptides (300 ng) were separated on a PicoFrit^TM^ Hypersil Gold aQ analytical column (1.9 μm, 75 μm id × 50 cm,) (New Objective). Muropeptides were eluted for 10 min with water + 0.1 % (*v*/*v*) formic acid + 0.8 % (*v*/*v*) acetonitrile (buffer A) and then with a 50-min acetonitrile linear gradient (0 to 22.5 %) in buffer A at a flow rate of 0.3 μl/min. The mass spectrometer was operated in standard data-dependent acquisition mode controlled by Xcalibur 2.2. The instrument was operated with a cycle of one MS (in the Orbitrap) acquired at a resolution of 120,000 at *m*/*z* 400 from 150 to 2000 *m*/*z* and the top 5 most abundant multiply charged (2+ and higher) ions in a given chromatographic window were subjected to either HCD fragmentation (isolation window 3 *m*/*z*, normalized collision energy = 25, activation time 10 ms) or ETD fragmentation (isolation window 3 *m*/*z*, normalized collision energy = 35, activation time 300 ms) in the linear ion trap with supplemental activation enabled. An FTMS target value of 1e6, an HCD target value of 50,000 and an ion trap MSn target value of 10,000 were used. Dynamic exclusion was enabled with a repeat duration of 30 s with an exclusion list of 500 and exclusion duration of 30 s. Lock mass of 445.120025 was enabled for all experiments.

#### Data analysis

We used Byonic version 2.8.0 to identify peptidoglycan forms, Byologic version 2.4.21 to compute extracted ion chromatograms (XICs) and relative abundances, and Byomap 2.4.21 to view and annotate peaks in the total ion chromatogram (TIC). We started by searching for PG monomers using a FASTA database containing only the peptide sequences AEM, AEMA and AEMG. We specified a fixed modification of +41.0443 on methionine (M) so that modified M represented mDAP with mass 172.0848 (=131.0405 + 41.0443). We enabled variable modifications of 277.116 (MurNAc), 396.174 (GlcN-deacetyl-MurNAc), 420.174 (GlcN-anhydro-MurNAc), 438.185 (GlcN-MurNAc) and 480.196 (GlcNAc-MurNAc) on the peptide N-terminus. We also enabled variable modifications of –0.984 (amidation) on E and mDAP. In a subsequent search, we turned off amidation, anhydro and deacetylation and used a wildcard modification of mass –130 to +210 Da to find unanticipated modifications and sequence variants. All searches after some initial exploratory searches used a “Manual score cut” of zero, 5 ppm precursor mass tolerance, 20 ppm fragment mass tolerance for HCD spectra and 0.5 Da fragment mass tolerance for ETD spectra.

We searched for PG dimers two different ways: cross-link search on a monomer database and “ordinary” search on a dimer database. Byonic includes automatic cross-link search with user-defined cross-link mass and residue specificity, but with the limitation that the cross-link connects two residues of the same type. We specified −18.0106 as the cross-link mass and M (meaning mDAP) as the residue type. We ran Byonic on a FASTA database of monomer sequences and enabled the glycans GlcN-MurNAc and GlcNAc-MurNAc on the N-terminus. Byonic then predicts the correct b/y (or c/z) ions for 3–3 linkages from Glycan-A-E-mDAP to any Glycan-A-E-mDAP-X (or –X-X) peptide, but slightly incorrect b/y (or c/z) ions for 4–3 linkages from Glycan-A-E-mDAP-A to Glycan-A-E-mDAP-X peptides, because it fails to predict the b-ion containing Glycan-A-E-mDAP and the complementary y-ion containing A and Glycan-A-E-mDAP-X. We also searched for dimers using a FASTA database containing concatenated sequences such as AEMAEMA and allowing glycan modifications on both N- and C-termini; this method predicts the correct ions for cleavages of peptide bonds up to the cross-link, that is, correct b1, b2, b3 and y6, y5, y4 for the 3–3 link, but incorrect ions for cleavages after the cross-link. Incorrect ion predictions adversely affect Byonic scores, and this is one of the reasons that we used a manual score cutoff of zero (the other reason is that the usual methods for creating decoy databases, for example, reversing peptide sequences, do not work well for PG analysis, in which accurate precursor mass may be more informative than fragmentation peaks, many of which are common to multiple scored candidates).

Byonic does not currently offer cross-link search for more than two linked peptides, so we searched for trimers using a FASTA database containing sequences such as AEMAEMAEMA and allowing glycan modifications on N- and C-termini as well as on M. As above, this method predicts correct ions for cleavages up to the first cross-link but incorrect ions after that. When searching for dimers and trimers, we did not search for all possible combinations of monomers, but limited attention to those in which all donor peptides are either AEM or AEMA, that is, the peptides acted upon by the l,d and d,d transpeptidases. We tried all the monomers we discovered in the monomer searches as the terminal acceptor peptide for dimers and trimers.

Byologic and Byomap offer complementary ways to view and quantify peptides, by XIC and TIC or UV trace, respectively. We used Byologic to validate and quantify PG forms identified by Byonic and Byomap to obtain a TIC trace, inspect MS full scans and map PG forms to TIC elution peaks. We used manual analysis to distinguish 3–3 from 4–3 linkages for the dimers containing one Glycan-A-E-mDAP and one Glycan-A-E-mDAP-A or two Glycan-A-E-mDAP-A’s, which is isobaric with a Glycan-A-E-mDAP and Glycan-A-E-mDAP-A-A dimer.

## Results and discussion

As shown in Tables 1, 2 and 3, the Byonic searches found 29.2, 57.4 and 13.4 % monomers, dimers and trimers, as percentages of the total of identified XIC quantities. These proportions are consistent with the percentages of 35.1, 56.6 and 8.3 % reported previously [11] based on UV traces quantifications and MALDI-TOF offline analyses rather than LC-ESI quantitation. We find that 77.6 % of monomers are GlcN-MurNAc-AEmA (here m = mDAP), in close agreement with 74.9 % from Peltier et al. These monomers containing tetrapeptide stems result from carboxypeptidase activity (see Electronic Supplementary Material (ESM) Fig. S2). GlcN-MurNAc-AEm is not the most common monomer because it is usually linked rather than free. We find that 42.7 % of dimers are GlcN-MurNAc-AEm linked to GlcN-MurNAc-AEmA, in close agreement with 43.8 % from Peltier et al. Our number, however, rises to 53.7 % if we include forms missing GlcN that, due to almost exactly correlated co-elution, we judge to be produced within the electrospray source. In-source decay also occurs with MALDI, but is harder to judge without elution profiles. The most common trimer contains two GlcN-MurNAc-AEm and one GlcN-MurNAc-AEmA, accounting for 30.7 % of all trimers (34.9 % including in-source decay) and 42.7 % for Peltier et al. We did not find anhydrous MurNAc as reported by Peltier et al., but rather anhydrous mDAP in the C-terminal position (see spectra on page 18 of the Fig. S3 in ESM), but this may be an in vitro water loss.

**Table 1:**
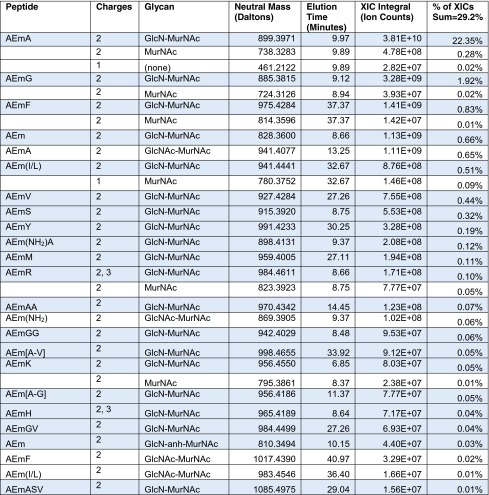
List of detected monomers with peptides (m = mDAP), glycans, precursor masses, apex elution times, XIC integrals and percent of reported XIC integrals

The XIC integral is the ion count for the monoisotopic precursor, summed over charge states for the monomers observed in more than one charge state, that is, AEmR and AEmH. Rows with peptides left blank show monomers formed in the electrospray source by loss of monosaccharides, recognizable as in-source decay by exact co-elution. Shaded rows show monomers with the most common glycan (GlcN-MurNAc). Brackets as in [A-G] indicate uncertain order of the amino acid residues; (I/L) indicates either isoleucine or leucine. 77.6 % of all monomers are GlcN-MurNAc-AEmA. Of the peptide sequence variants, only AEmG has been reported before

**Table 2:**
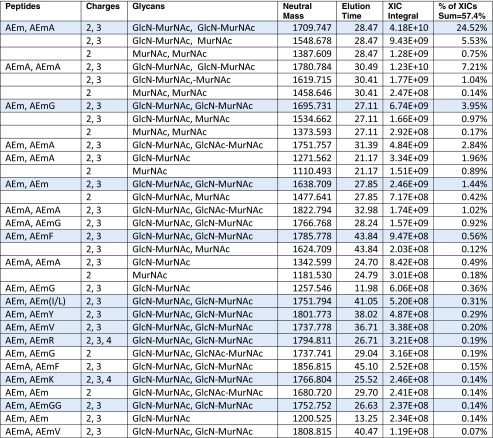
List of most abundant dimers with peptides (m = mDAP), glycans, masses, apex elution times and abundances

Shaded rows show dimers with the most common donor peptide (AEm) and glycans (GlcN-MurNAc); acceptor peptide prevalences (AEmA, AEmG, AEm, AEmF, etc.) agree well with monomer abundances. About 54 % of the dimers correspond to GlcN-MurNAc-AEm donors cross-linked to GlcN-MurNAc-AEmA acceptors via a 3–3 bond

**Table 3:** List of most abundant trimers with glycans, precursor masses, apex elution times and abundances

Peptides	Charges	Glycans	Neutral mass	Elution time	XIC integral	% of XICs sum = 13.4 %
AEm, AEm, AEmA	2, 3, 4	GlcN-MurNAc, GlcN-MurNAc, GlcN-MurNAc	2520.096	34.76	7.03E + 09	4.12 %
	3, 4	GlcN-MurNAc, MurNAc, GlcN-MurNAc	2359.027	34.84	9.39E + 08	0.55 %
AEm, AEmA, AEmA	2, 3, 4	GlcN-MurNAc, GlcN-MurNAc, GlcN-MurNAc	2591.133	35.77	4.48E + 09	2.63 %
AEm, AEm, AEmA	2, 3, 4	GlcN-MurNAc, GlcN-MurNAc	2081.911	31.87	1.50E + 09	0.88 %
AEm, AEm, AEmA	2, 3	GlcN-MurNAc, GlcN-MurNAc, GlcNAc-MurNAc	2562.107	36.79	1.38E + 09	0.81 %
AEm, AEm, AEmG	2, 3, 4	GlcN-MurNAc, GlcN-MurNAc, GlcN-MurNAc	2506.080	33.21	1.19E + 09	0.70 %
AEm, AEmA, AEmA	2, 3	GlcN-MurNAc, GlcN-MurNAc, GlcNAc-MurNAc	2633.144	37.54	1.14E + 09	0.67 %
AEmA, AEmA, AEmA	3, 4	GlcN-MurNAc, GlcN-MurNAc, GlcN-MurNAc	2662.170	36.95	8.39E + 08	0.49 %
AEm, AEmA, AEmA	2, 3	GlcN-MurNAc, MurNAc, GlcN-MurNAc	2430.064	36.87	7.07E + 08	0.41 %
AEm, AEm, AEmA	3, 4	GlcNAc-MurNAc, GlcNAc-MurNAc, GlcNAc-MurNAc	2646.138	35.69	5.05E + 08	0.30 %
AEm, AEmA, AEmG	3	GlcN-MurNAc, GlcN-MurNAc, GlcN-MurNAc	2577.117	34.00	4.77E + 08	0.28 %
AEmA, AEmA, AEmG	3, 4	GlcNAc-MurNAc, MurNAc, GlcNAc-MurNAc	2571.107	40.13	3.88E + 08	0.23 %
AEm, AEmA, AEmG	3	GlcNAc-MurNAc, MurNAc, GlcNAc-MurNAc	2500.070	39.34	3.87E + 08	0.23 %
AEm, AEm, AEm	3, 4	GlcN-MurNAc, GlcN-MurNAc, GlcN-MurNAc	2449.059	33.68	3.45E + 08	0.20 %
AEm, AEm, AEmA	2, 3, 4	GlcN-MurNAc	1643.726	26.29	3.43E + 08	0.20 %
AEmA, AEmA, AEmA	3	GlcN-MurNAc, GlcN-MurNAc, GlcNAc-MurNAc	2704.181	38.57	3.14E + 08	0.18 %
AEm, AEmA, AEmA	3, 4	GlcN-MurNAc, GlcN-MurNAc	2152.948	32.51	2.31E + 08	0.14 %
AEm, AEmA, AEmG	3	GlcN-MurNAc, GlcN-MurNAc, GlcNAc-MurNAc	2619.128	35.54	1.99E + 08	0.12 %
AEm, AEm, AEmG	3	GlcN-MurNAc, GlcNAc-MurNAc, GlcN-MurNAc	2548.091	34.46	1.82E + 08	0.11 %
AEm, AEm, AEmG	3	GlcN-MurNAc, MurNAc, GlcN-MurNAc	2345.012	33.21	1.54E + 08	0.09 %

35 % of the trimers correspond to a GlcN-MurNAc-AEm donors cross-linked to GlcN-MurNAc-AEmA acceptor stems via 3–3 bonds

**Fig. 2:**
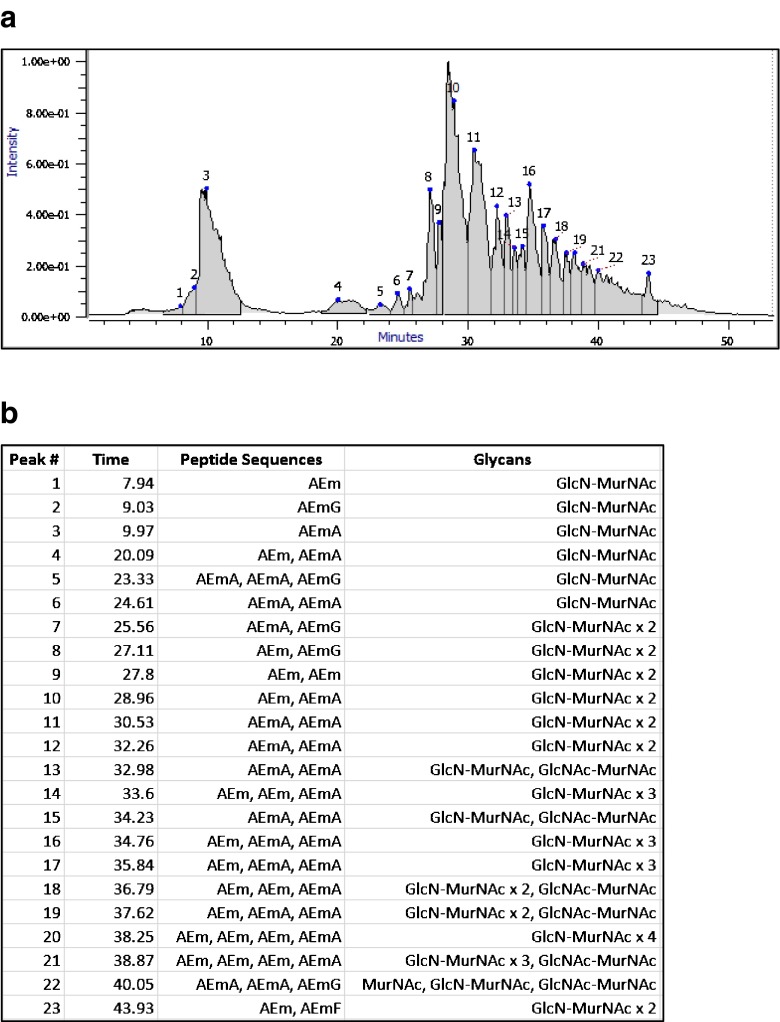
(**a**) Total ion chromatogram shows PG components ordered by hydrophobicity, monomers generally eluting before dimers and trimers. (**b**) Major component for each elution peak. Most peaks also contain several minor components. Peaks 11 and 12 and also 16 and 17, contain isomers, possibly epimers, indistinguishable by precursor mass and MS/MS scans

As shown in Fig. 2, we found at least one identification for each distinct peak in the TIC elution profile. Byomap computes a summed MS spectrum for each elution peak, and in almost all cases, Fig. 2 lists the identification matching the most intense peak in the MS spectrum. An exception is elution peak 14, in which the most intense spectral peak has neutral mass 1707.731 and remains unidentified. The MS spectra for elution peaks 21 and 22 contain numerous low abundance forms, most but not all of which match masses from Tables 2 and 3.

**Fig. 3:**
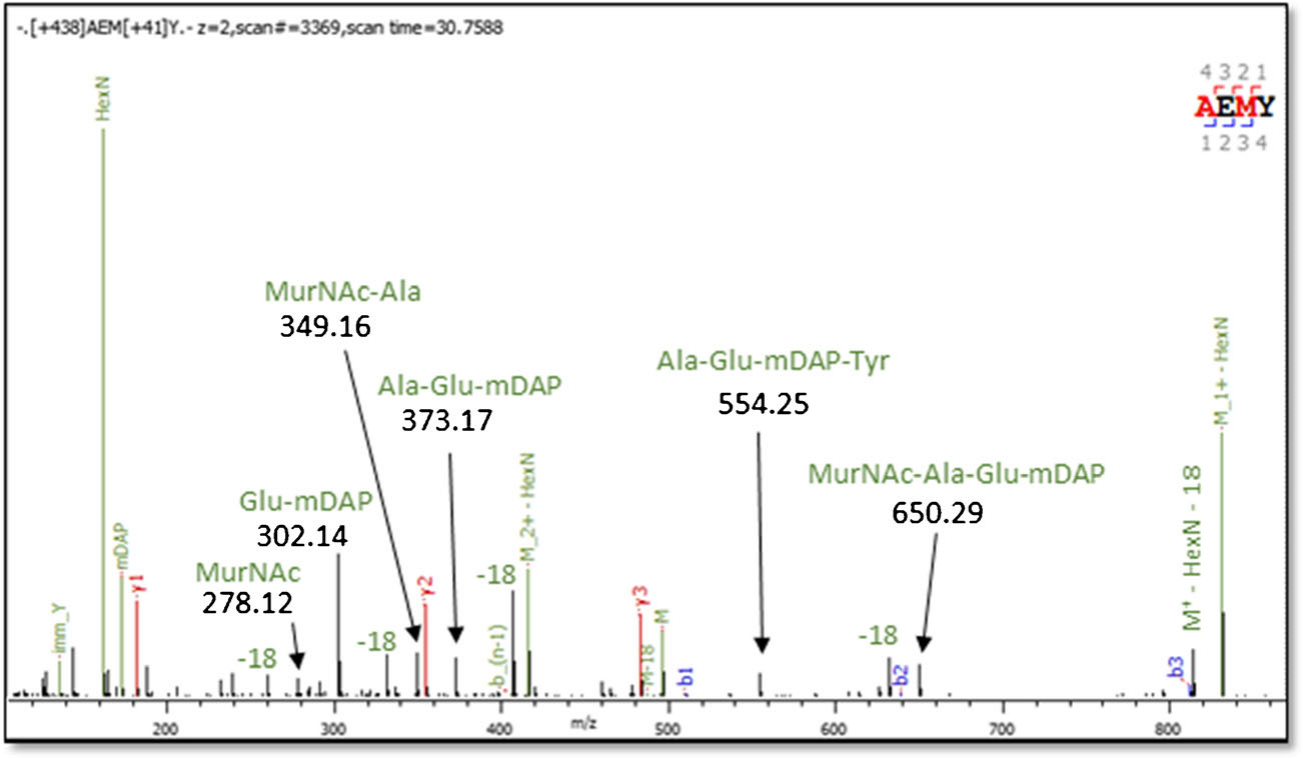
Collisional dissociation (HCD) spectrum of an unanticipated sequence variant, recognized by high-accuracy (5 ppm precursor and 20 ppm fragment) mass spectrometry. Other fourth residue variants include: Phe, Ile/Leu, Ser, Met, Val, Arg, Lys and His. The small peptide fragmentation diagram on the right side of the tandem mass spectrum shows Byonic’s automatic assignment of spectrum peaks

The most obvious departure from previous results is the discovery of a number of peptide sequence variants, including 5- and even 6-residue peptides, which have not been reported before. Their presence in *C. difficile* is likely to be due to the exchange activity of l,d-transpeptidases (Fig. S2 in the ESM), which has been demonstrated in vitro [12] for several amino acids. These sequence variants are evidenced by precursor masses matching within 5 ppm, in most cases within 1 ppm, as well as high-quality fragmentation spectra, one of which is shown Fig. 3 and others in Fig. S3 in the ESM. The elution times of the monomers, with Arg, Lys, His and Ser giving earlier elution times than Gly and Ala, and Met, Val, Tyr, Lys and Phe giving later elution times, matches measurements made for peptide elution time predictors [13]. It is tempting to assume that such unconventional muropeptides are present in other bacterial peptidoglycans but have not been reported so far due to their low abundance. Both high MS sensitivity and automated MS/MS spectrum analysis will be required to test if similar unconventional muropeptides are found in other PG.

It is worth noting that our nano-LC strategy uses only 300 ng of PG material, about 30 times less material than a recently published “highly sensitive” UPLC strategy [6]. The relatively short run time (about 50 min) is much faster than traditional offline fractionation (140 min for *C. difficile*), and comparable to the UPLC strategy [6], which employs no mass spectrometry and is hence more suited to comparison than to discovery. Nano-LC-ESI-MS/MS thus appears to be the most effect data acquisition strategy yet devised for PG structural analyses.

Complementing the data acquisition strategy, our bioinformatics search strategy gives high sensitivity and acceptably low false-positive rate, even for trimers for which almost 2/3 of the fragment peak predictions are incorrect. The primary reason for the low error rate is the simplicity of the search relative to other glycoproteomics searches. The trimer search includes about 2 × 2 × 20 peptide sequences (two possibilities for each donor peptide and up to 20 for the terminal acceptor peptide) and 5 × 5 × 5 possibilities for glycosylation (no glycan, MurNAc, GlcN-MurN, GlcN-MurNAc and GlcNAc-MurNAc), for a total of about ∼5000 candidates with less than 500 distinct masses. Accurate precursor mass alone, without fragmentation, can resolve almost all precursors in a “search space” of this size; for example, GlcNAc-MurNAc-AEmA and GlcN-MurNAc-AEM(I/L) differ by 38 ppm, almost 8 times the precursor mass tolerance of 5 ppm. We also find that our search strategy gave acceptably low false-negative rate: All the TIC elution peaks in Fig. 2 have identifications, and in all but a few cases mentioned above, these identifications explain the major peaks in the summed MS spectra.

**Fig. 4:**
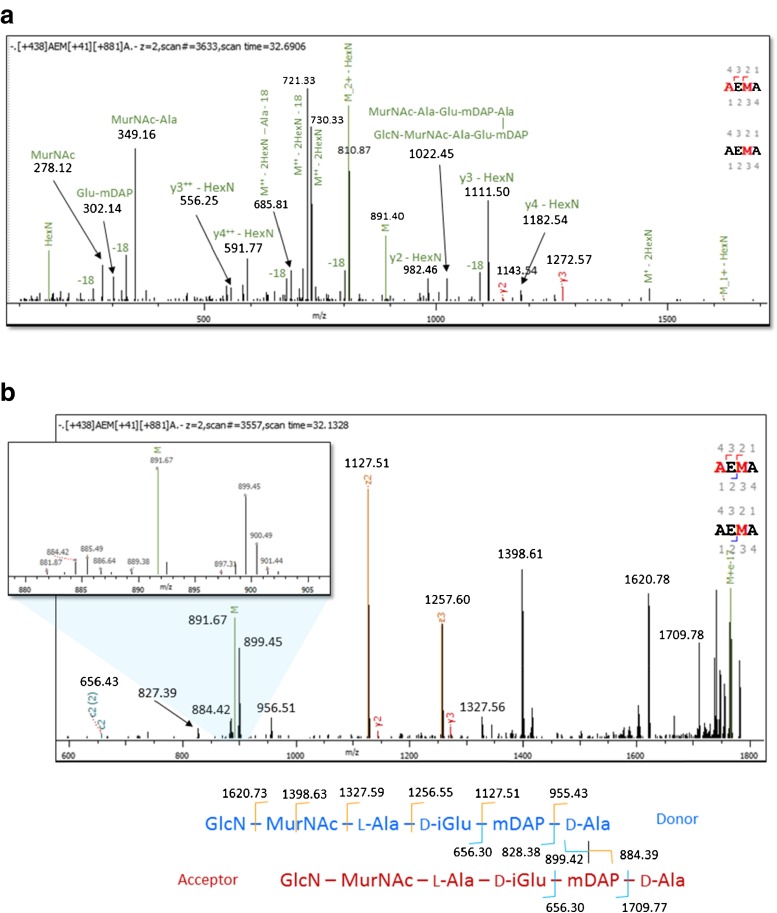
(**a**) HCD and (**b**) ETD spectra of a dimer with that could be either GlcN-MurNAc-A-E-mDAP 3–3 linked to GlcN-MurNAc-A-E-mDAP-A-A or GlcN-MurNAc-A-E-mDAP-A 4–3 linked to GlcN-MurNAc-A-E-mDAP-A. The HCD spectrum contains no peaks (such as an A-A y-ion for 3–3) that can distinguish the two possibilities, but the ETD spectrum contains peaks at 899.45 and 884.42 matching the theoretical masses (given on the fragmentation diagram) of 899.32 and 884.39 for c- and z-ions splitting donor and acceptor peptides. The ETD spectrum therefore shows that the dimer is 4–3 linked. Byonic gives a fragmentation diagram and annotates c2, y2, y3, z2 and z3 for [+438]AEM[+41][+881]A crosslinked to [+438]AEM[+41]A, where [+438] represents the disaccharide, M[+41] represents mDAP, and [+881] the cross-link “modification” on M[+41], but Byonic does not annotate internal fragments nor the disambiguating peaks at 899.45 and 884.42

As mentioned above, we relied on manual analysis to distinguish 3–3 from 4–3 cross-links, so we limited attention to the most abundant dimers. All the MS/MS spectra we examined of precursors with mass matching 2 × GlcN-MurNAc-AEmA appeared to contain 4–3 cross-links, rather than a 3–3 link between GlcN-MurNAc-AEm and GlcN-MurNAc-AEmAA. This finding is of course unsurprising because it agrees with monomer abundances. The sample may, however, contain some amount of the 3–3 form co-eluting with the 4–3 form. Figure 4 gives an example manual analysis for both HCD and ETD spectra. The HCD spectrum contains no peaks distinguishing 3–3 and 4–3 cross-links, partly because HCD spectra of PG components are dominated by internal fragments, few of which can be unambiguously linked to one cross-link form. For example, an internal fragment with the mass of two mDAP residues would be unambiguous evidence of a 3–3 link, but an internal fragment with the mass equal to the sum of one Ala and two mDAP residues could arise from either cross-link form. ETD spectra, however, are much simpler, dominated by c- and z-ions, and we found that ETD spectra of PG dimers generally include strong peaks such as 899.45 and 884.42 in Fig. 4 for the c- and z-ions splitting the monomers. We find that ETD, which to our knowledge has not been applied to PG analysis before, offers a promising alternative or adjunct to collisional fragmentation.

**Fig. 5:**
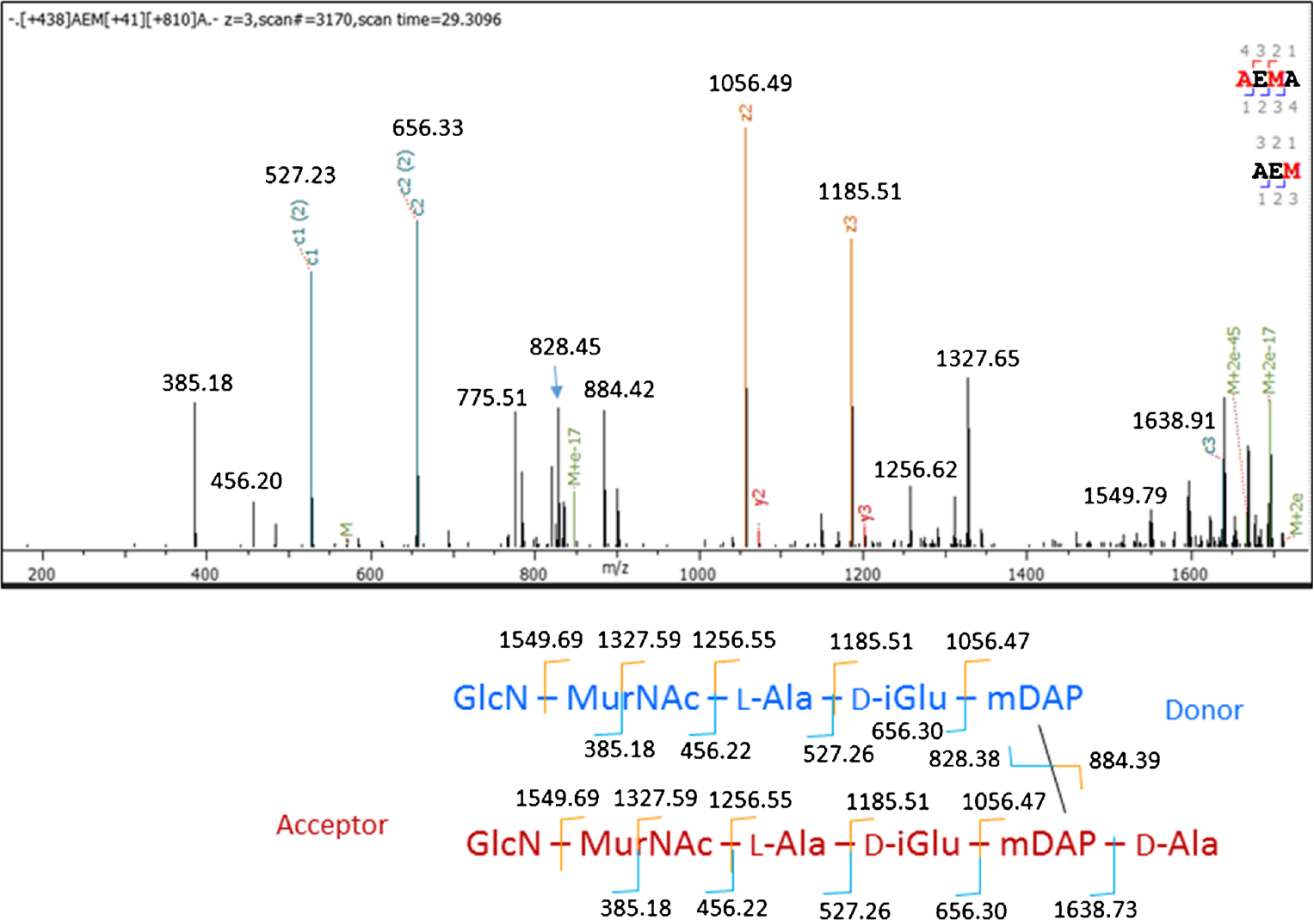
ETD spectrum of GlcN-MurNAc-A-E-mDAP 3–3 linked to GlcN-MurNAc-A-E-mDAP-A. The peaks at 828.45 and 884.42 match the theoretical masses of 828.38 and 884.39 for c- and z-ions splitting donor and acceptor peptides. ETD fragments PG components at both peptide and glycosidic bonds, including the C–O bond between the lactyl group and MurNAc, shown in the fragmentation diagram with a line across MurNAc

All the MS/MS spectra we examined of dimers containing GlcN-MurNAc-AEm and GlcN-MurNAc-AEmA appeared to contain 3–3 cross-links. Figure 5 gives a manually annotated spectrum. Notice that in both Figs. 4 and 5, the ETD spectra show strong cleavage at the glycosidic bonds between GlcN and MurNAc (thereby localizing the deacetylation), between MurNAc and the lactyl group, and between the lactyl group and the alanine residue in position 1; this cleavage pattern is somewhat unexpected because ETD generally cleaves peptide bonds more readily than glycosidic bonds [14]. Also notice that in Fig. 4, the cleavage between mDAP and d-Ala shows a hydrogen transfer from the c-ion to the z-ion, a common occurrence in ETD spectra of peptides with precursor charge z = 2+ [15].

As shown in Figure S1 in the ESM, the 4–3 dimer shows two distinct elution peaks at 30.6 and 32.2 min, with the earlier peak about twice as abundant as the later. The 3–3 dimer has distinct peaks at 28.6 and 31 min in the ratio of about 4:1. In both cases, the MS/MS spectra show no differences between the two elution peaks. We speculate as others have before [11, 16] that these dimers have two forms each, differing only in stereochemistry.

## Conclusion

This study describes an effective data-dependent tandem mass spectrum acquisition strategy, along with an almost fully automated data analysis approach to identify muropeptide structures, employing *C. difficile* PG as a proof of concept. We showed that nano-LC separation combined with the low ppm *m*/*z* accuracy of the Orbitrap mass spectrometer allows the structural analysis of sub-microgram amounts of peptidoglycan extracted from the equivalent of as few as 10^4^ cells in less than an hour. The objective automated analysis of MS/MS spectra by the Byonic software confirmed previous structural analyses and revealed several modifications found in low abundance that had not been reported so far in *C. difficile*, in particular a wide variety of C-terminal amino acids in acceptor peptides. Taken together, we propose that this novel analysis pipeline could replace the conventional chromatographic and mass spectrometric approaches used for the past 30 years.

## Electronic supplementary material

Below is the link to the electronic supplementary material.

ESM 1 (PDF 890 kb)
