# Supplementary material for: Towards an automated analysis of bacterial peptidoglycan structure
Source: Anal Bioanal Chem. 2016 Aug 13;409(2):551–60. doi: 10.1007/s00216-016-9857-5 (PMC5203844; doi:10.1007/s00216-016-9857-5)
Supplement: Supplementary file 1 — (PDF 890 kb) [file 216_2016_9857_MOESM1_ESM.pdf]

Analytical and Bioanalytical Chemistry

Electronic Supplementary Material

Towards an automated analysis of bacterial peptidoglycan structure

Marshall Bern, Richard Beniston, Stéphane Mesnage

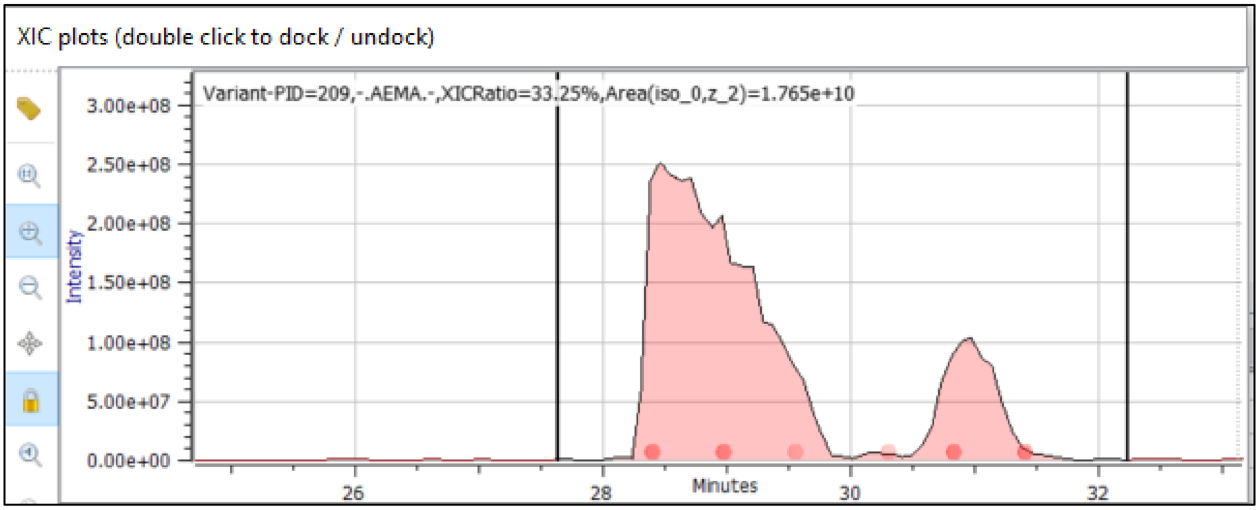

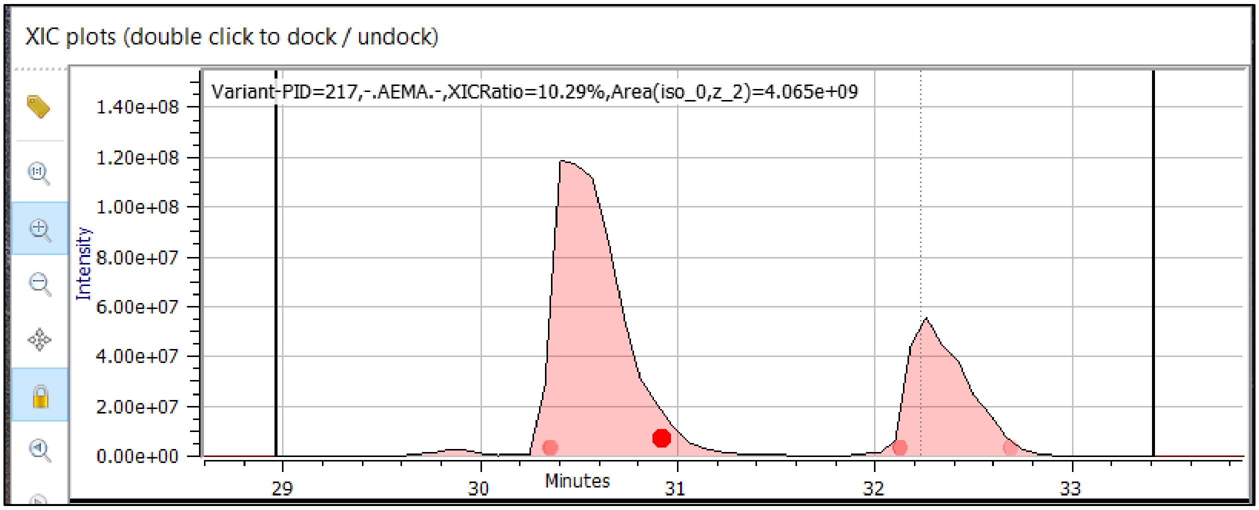

Fig. S1 Split Elution. Both AEm-3,3-AEmA (top) and AEmA-4,3-AEmA (bottom) show two distinct elution peaks. MS/MS spectra (scan times indicated by red dots) do not differ in any discernible way between the two elution pulses. XIC plots from Byologic (Protein Metrics Inc.)

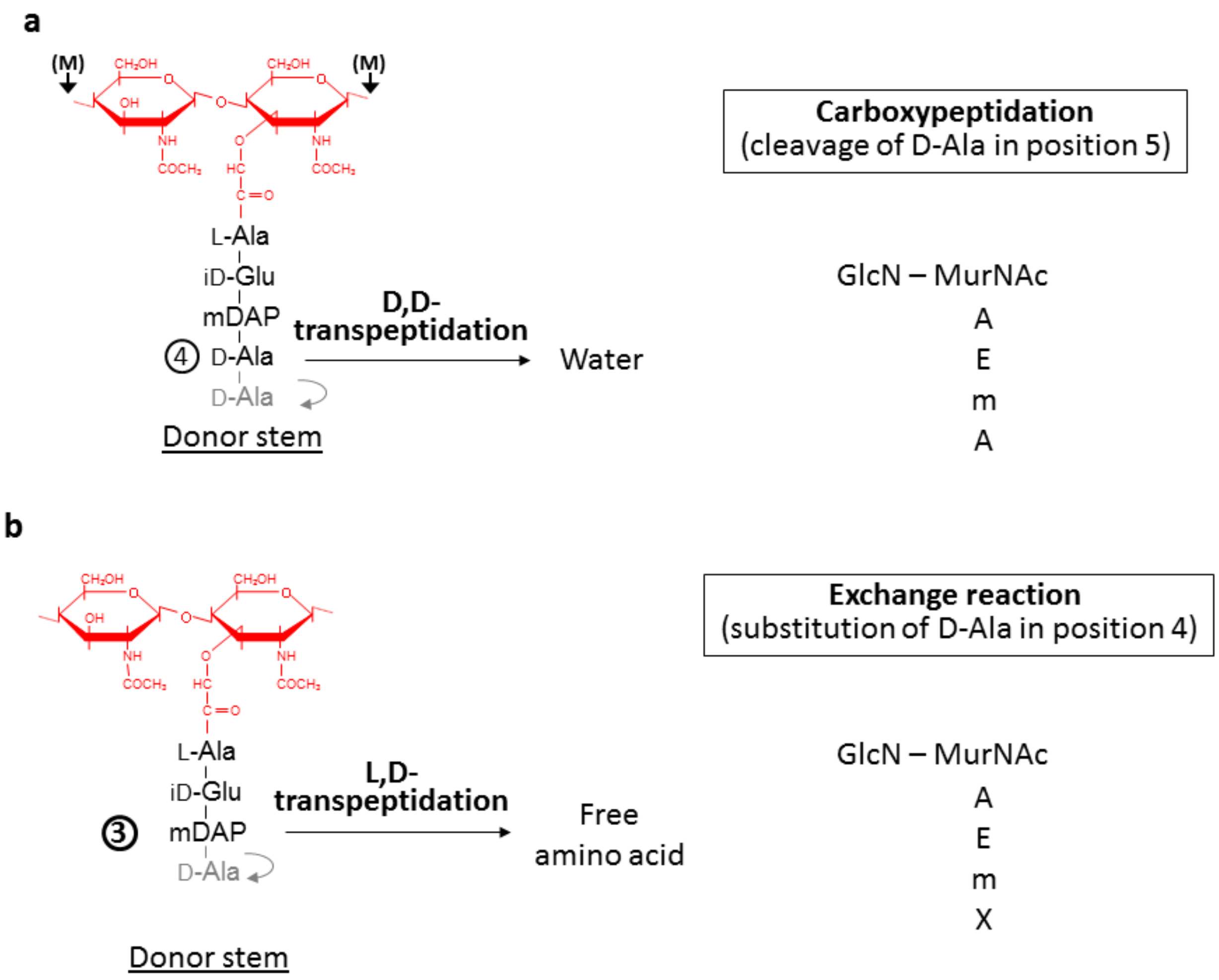

Fig. S2 Transpeptidation reactions. (a) Some low molecular weight D-D-transpeptidases display carboxypeptidase activity, and can generate disaccharide-AEmA monomers. Like high molecular weight D,D transpeptidases that polymerize PG, they act on disaccharide-AEmAA substrates as donor but use water as an acceptor molecule. (b) L-D-transpeptidases can exchange the D-Ala in position 4 of disaccharide-AEmA with a free amino acid, as shown by *in vitro* experiments (Mainardi et al, “A novel peptidoglycan cross-linking enzyme for a beta-lcatam-resistant transpeptidation pathway”, J. Biological Chemistry, 2005).

Fig. S3 The next 16 pages show HCD (collisional) and ETD (electron transfer) fragmentation spectra of peptide monomers with unusual amino acid residues, given in descending order of measured abundance, as shown in Table 1 in the main text. In the spectrum assignments, [+438] indicates GlcN-MurNAc, M[+41] is *meso*-diaminopimelic acid (mDAP), and M[+41][-41] is methionine. Spectra are shown as annotated by Byonic. Peaks annotated mDAP are internal fragments at 173.092 Da, the mass of the amino acid residue plus a proton

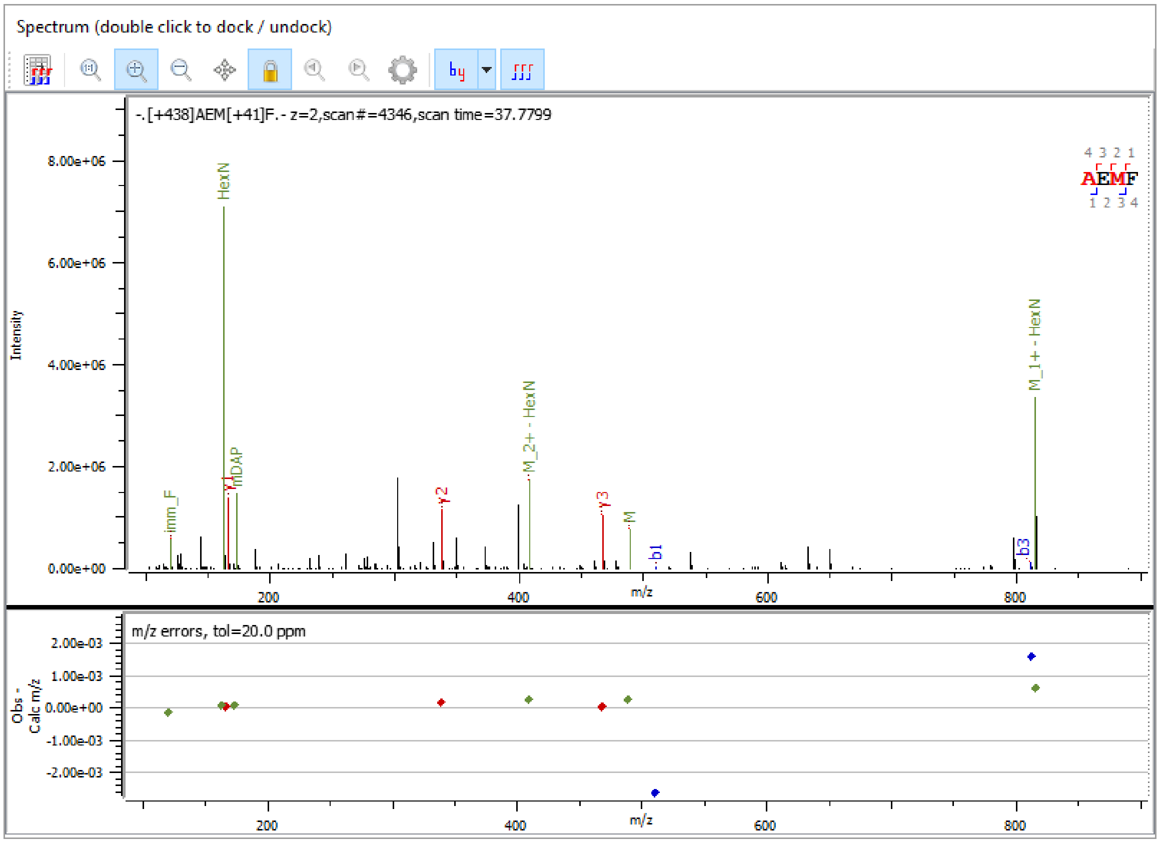
Spectrum (double click to dock / undock)
-.[+438]AEM[+41]F.- z=2,scan#=4346,scan time=37.7799
m/z errors, tol=20.0 ppm

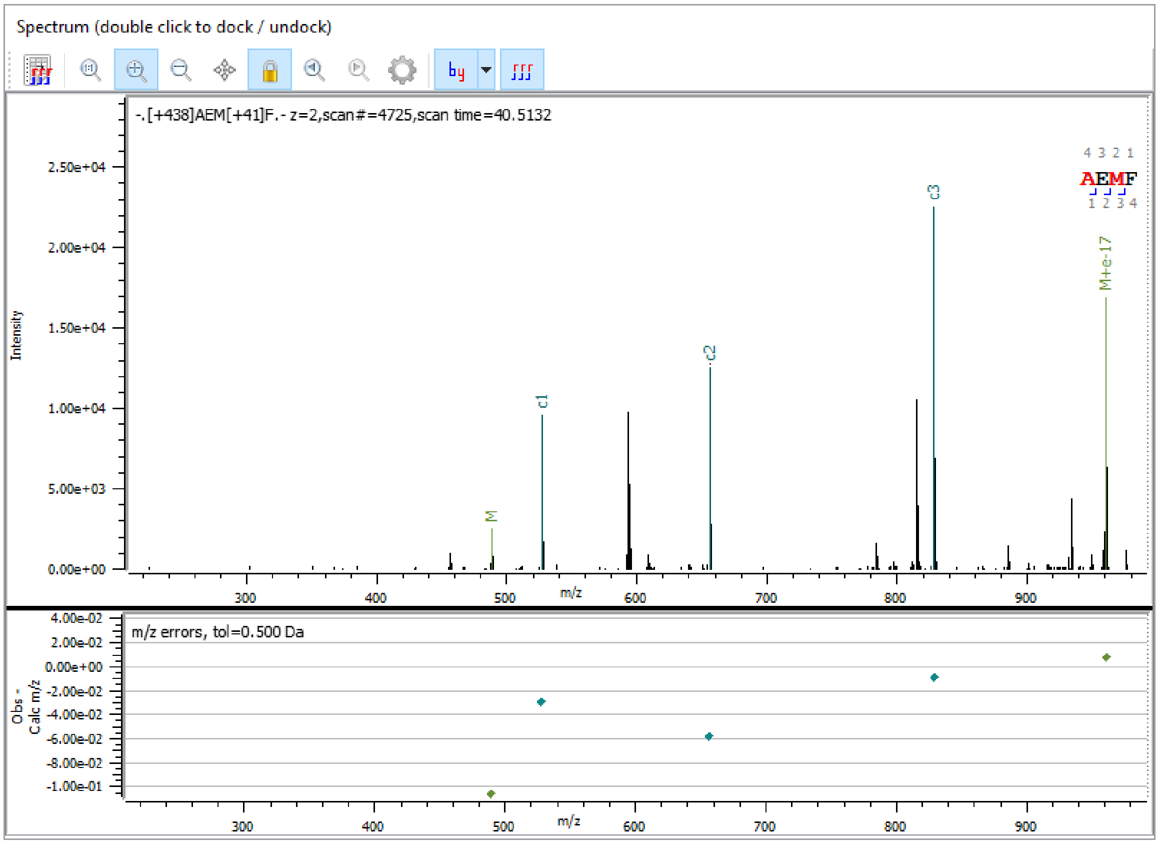
Spectrum (double click to dock / undock)
-.[+438]AEM[+41]F.- z=2,scan#=4725,scan time=40.5132
m/z errors, tol=0.500 Da

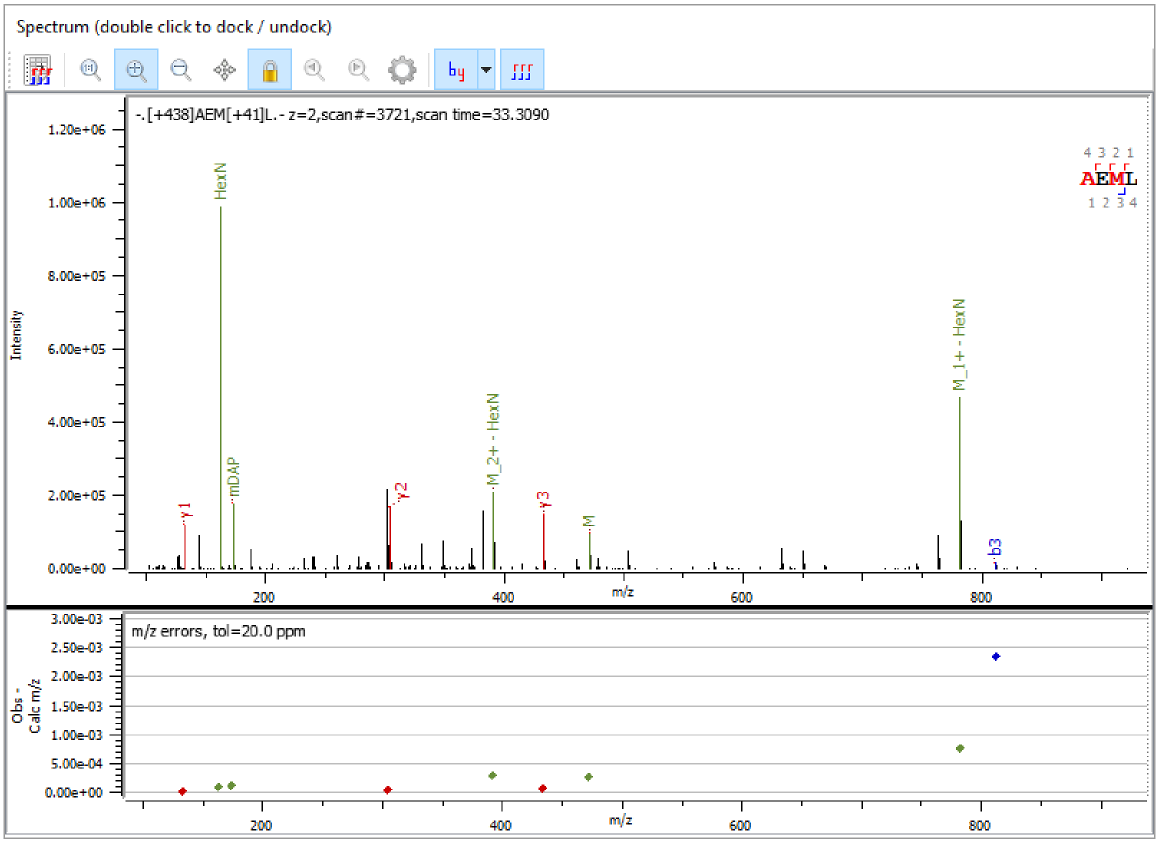
Spectrum (double click to dock / undock)
-.[+438]AEM[+41]L.- z=2,scan#=3721,scan time=33.3090
Intensity
HexN
mDAP
y1
y2
M_2+ - HexN
y3
M
M_1+ - HexN
b3
AEML
m/z
m/z errors, tol=20.0 ppm
Obs - Calc m/z

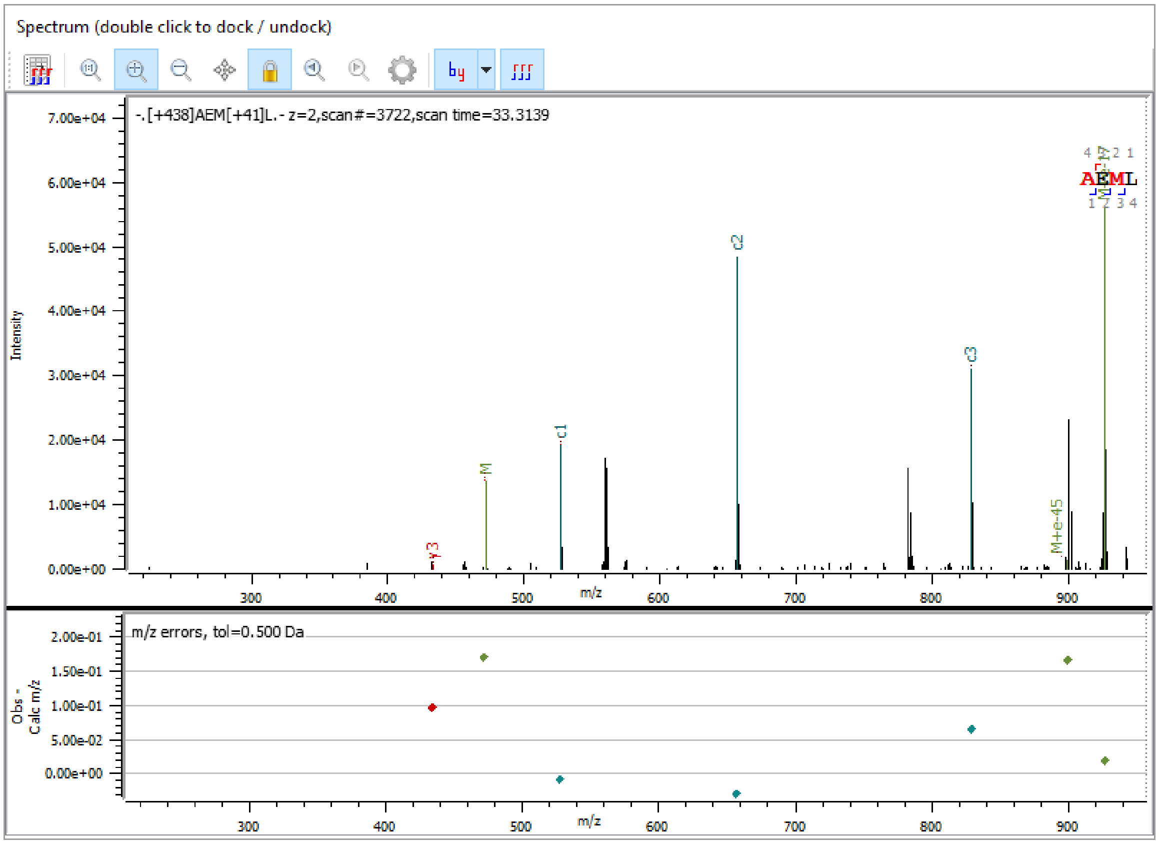
Spectrum (double click to dock / undock)
-.[+438]AEM[+41]L.- z=2,scan#=3722,scan time=33.3139
Intensity
y3
M
c1
c2
c3
M+e-45
AEML
m/z
m/z errors, tol=0.500 Da
Obs - Calc m/z

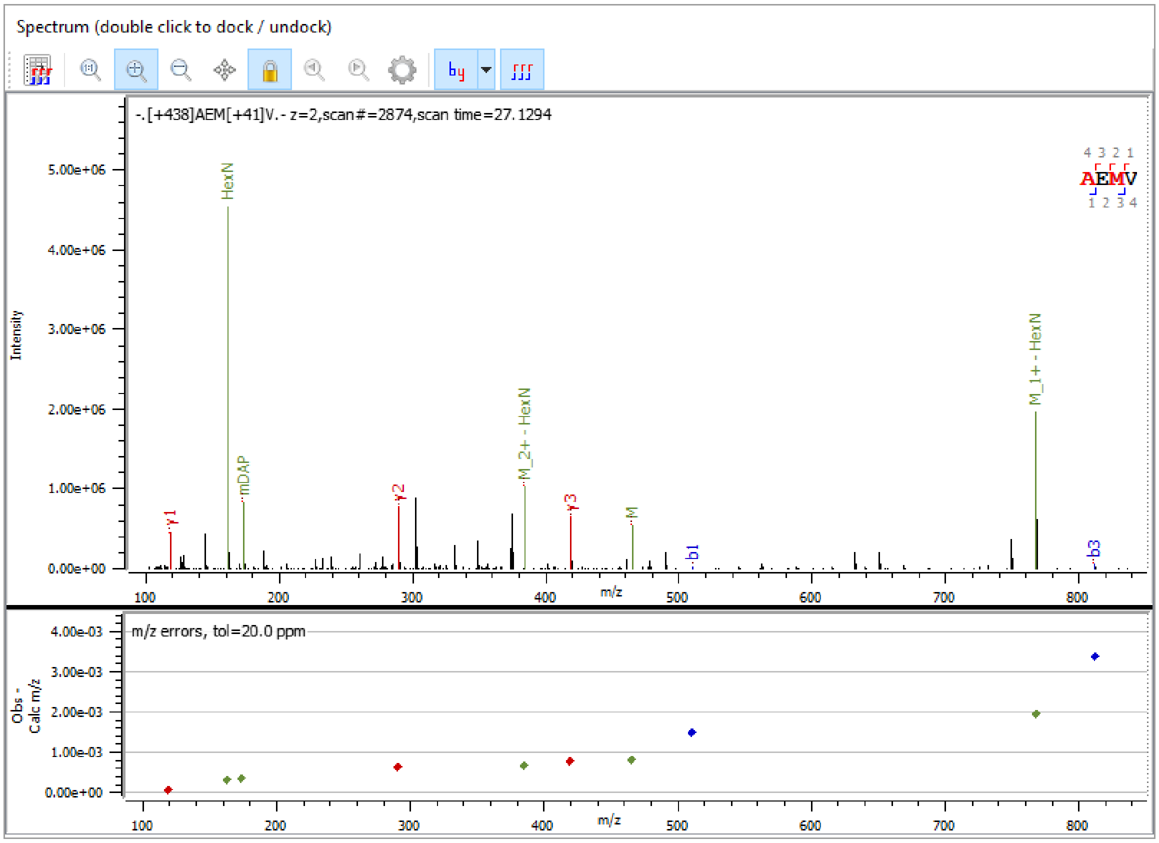
Spectrum (double click to dock / undock)
-.[+438]AEM[+41]V.- z=2,scan#=2874,scan time=27.1294
Intensity
m/z
HexN
mDAP
y1
y2
M_2+ - HexN
y3
M
b1
M_1+ - HexN
b3
AEMV
m/z errors, tol=20.0 ppm
Obs - Calc m/z

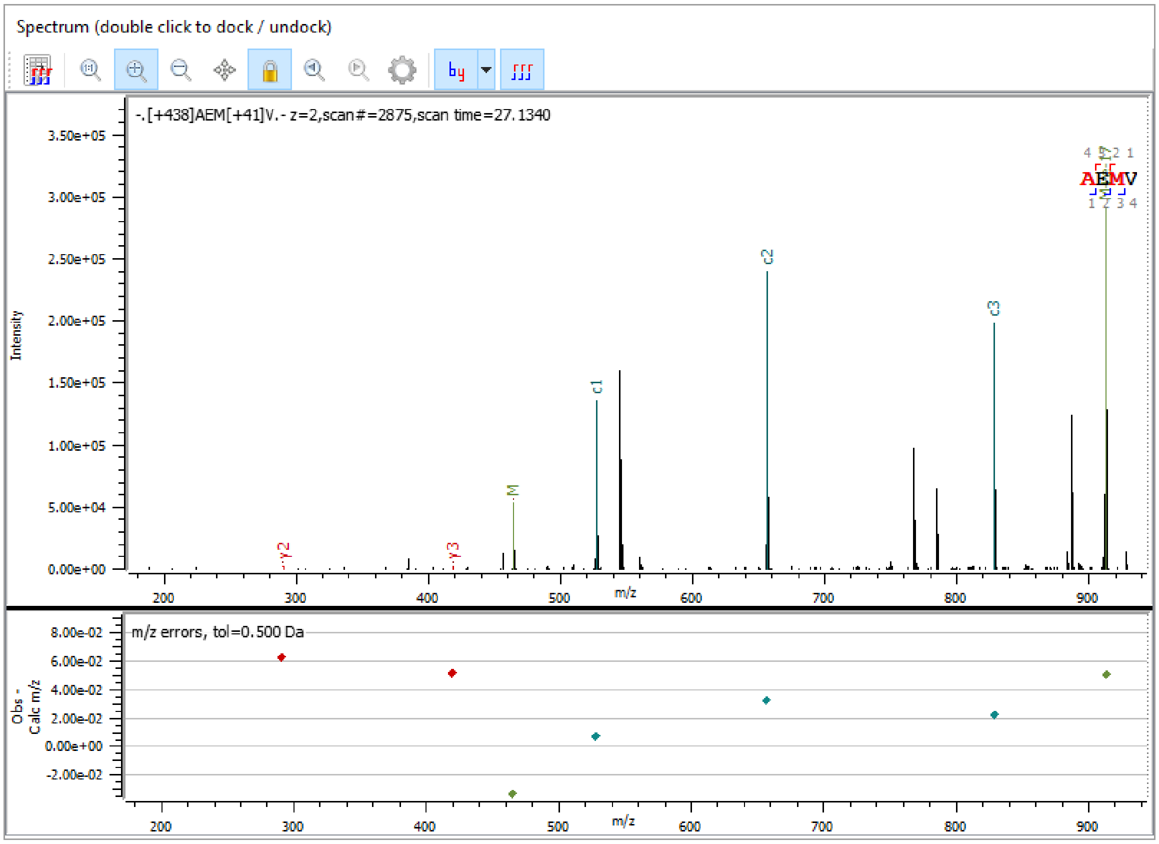
Spectrum (double click to dock / undock)
-.[+438]AEM[+41]V.- z=2,scan#=2875,scan time=27.1340
Intensity
m/z
y2
y3
M
c1
c2
c3
AEMV
m/z errors, tol=0.500 Da
Obs - Calc m/z

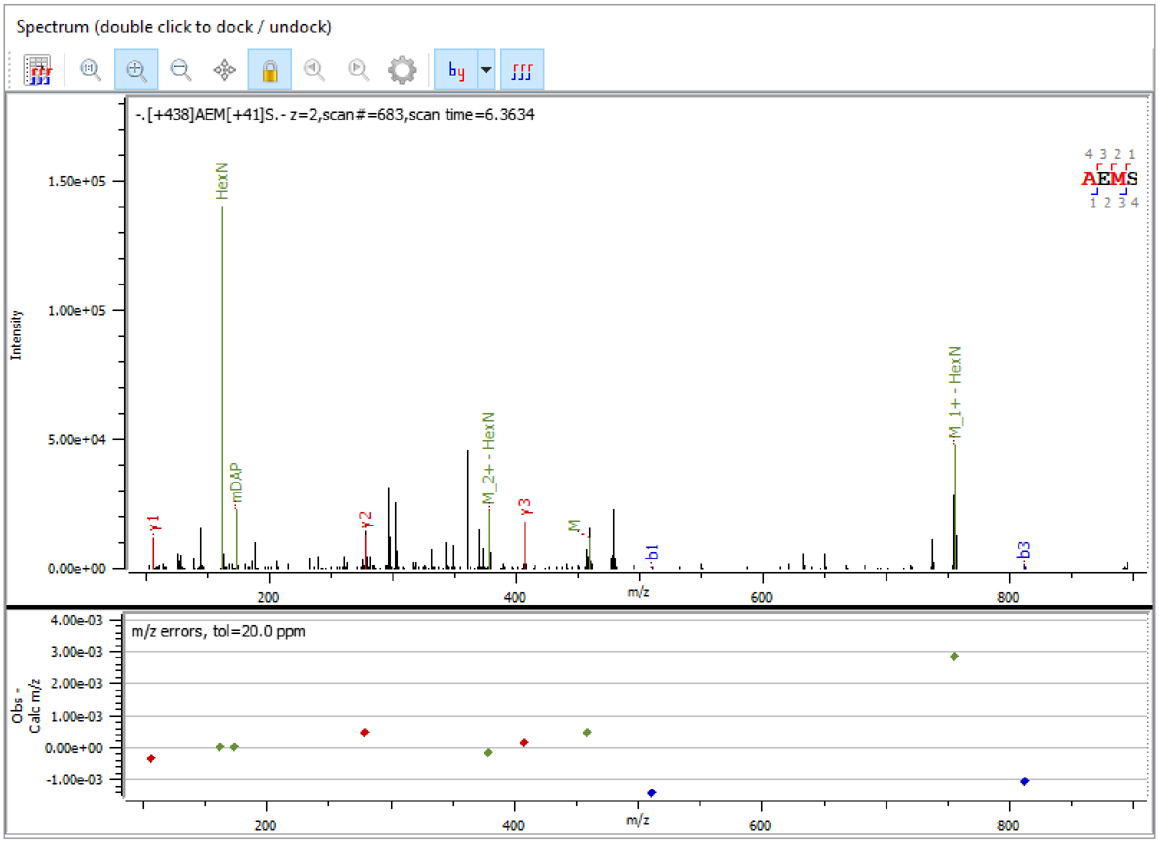
Spectrum (double click to dock / undock)
-.[+438]AEM[+41]S.- z=2,scan#=683,scan time=6.3634
AEMS
Intensity
HexN
mDAP
y1
y2
y3
M_2+ - HexN
M
b1
M_1+ - HexN
b3
m/z
m/z errors, tol=20.0 ppm
Obs - Calc m/z

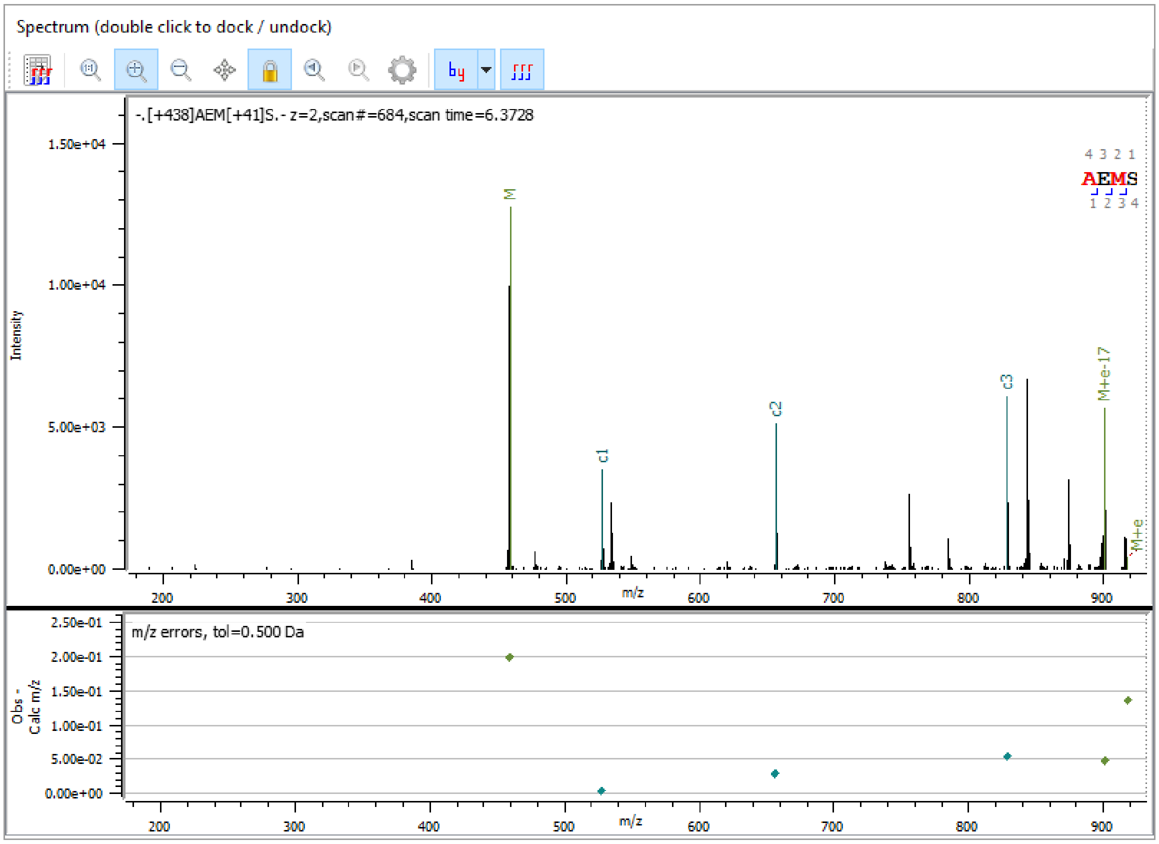
Spectrum (double click to dock / undock)
-.[+438]AEM[+41]S.- z=2,scan#=684,scan time=6.3728
AEMS
Intensity
M
c1
c2
c3
M+e-17
M+e
m/z
m/z errors, tol=0.500 Da
Obs - Calc m/z

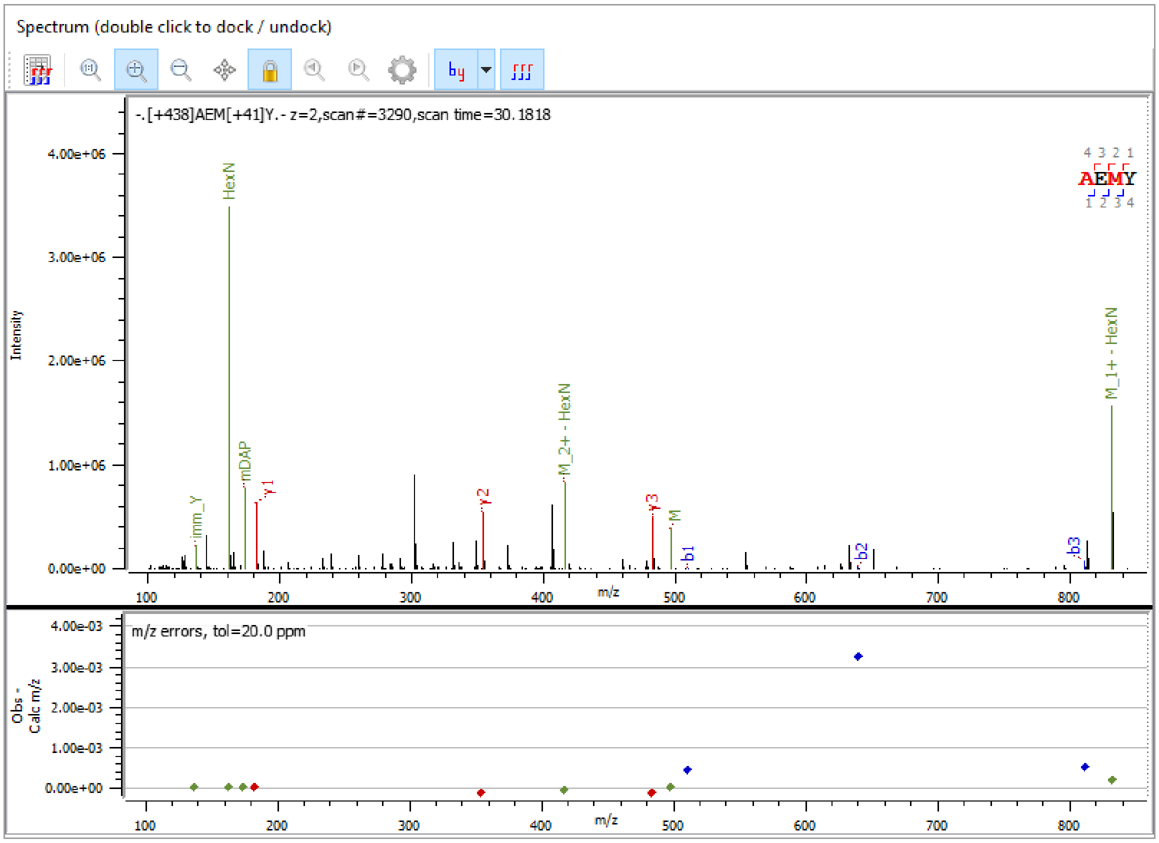
Spectrum (double click to dock / undock)
-.[+438]AEM[+41]Y.- z=2,scan#=3290,scan time=30.1818
AEMY
HexN
imm_Y
mDAP
y1
y2
M_2+ - HexN
y3
M
b1
b2
b3
M_1+ - HexN
Intensity
m/z
m/z errors, tol=20.0 ppm
Obs - Calc m/z

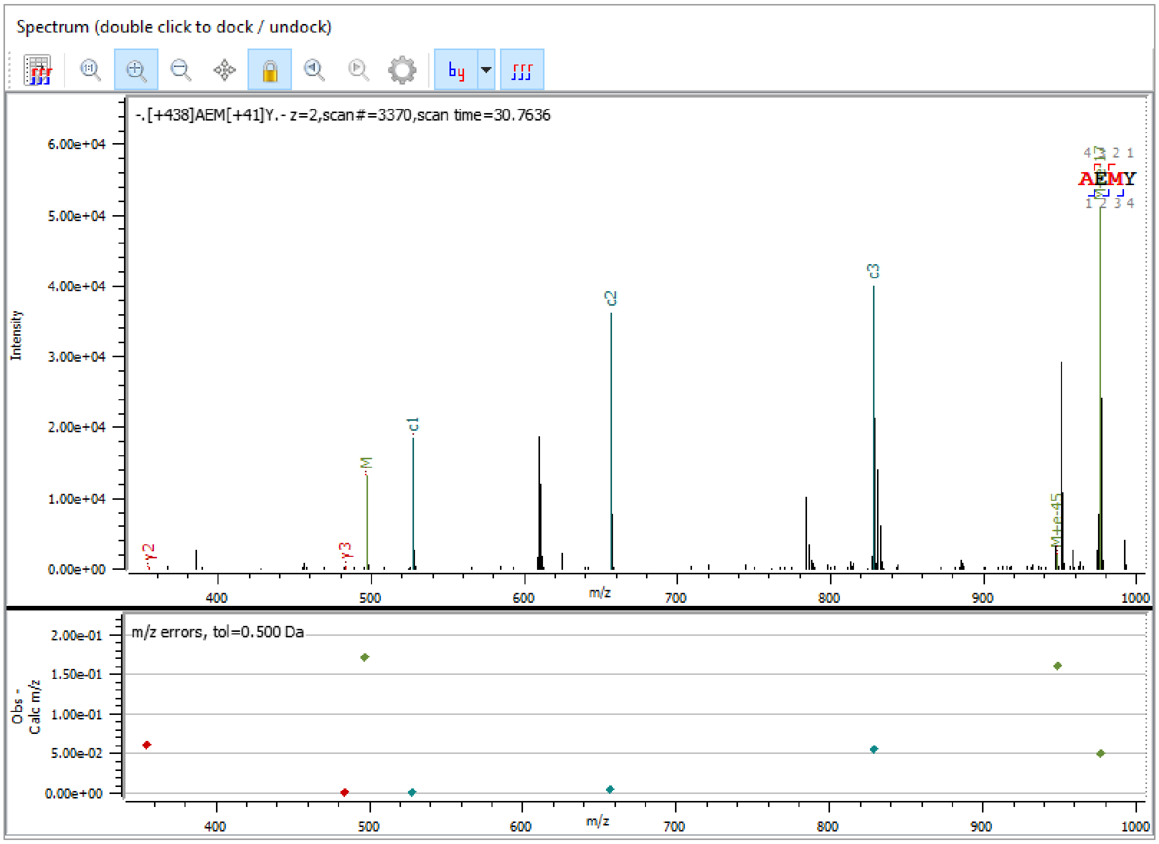
Spectrum (double click to dock / undock)
-.[+438]AEM[+41]Y.- z=2,scan#=3370,scan time=30.7636
AEMY
c3
c2
c1
M
y2
y3
Intensity
m/z
m/z errors, tol=0.500 Da
Obs - Calc m/z

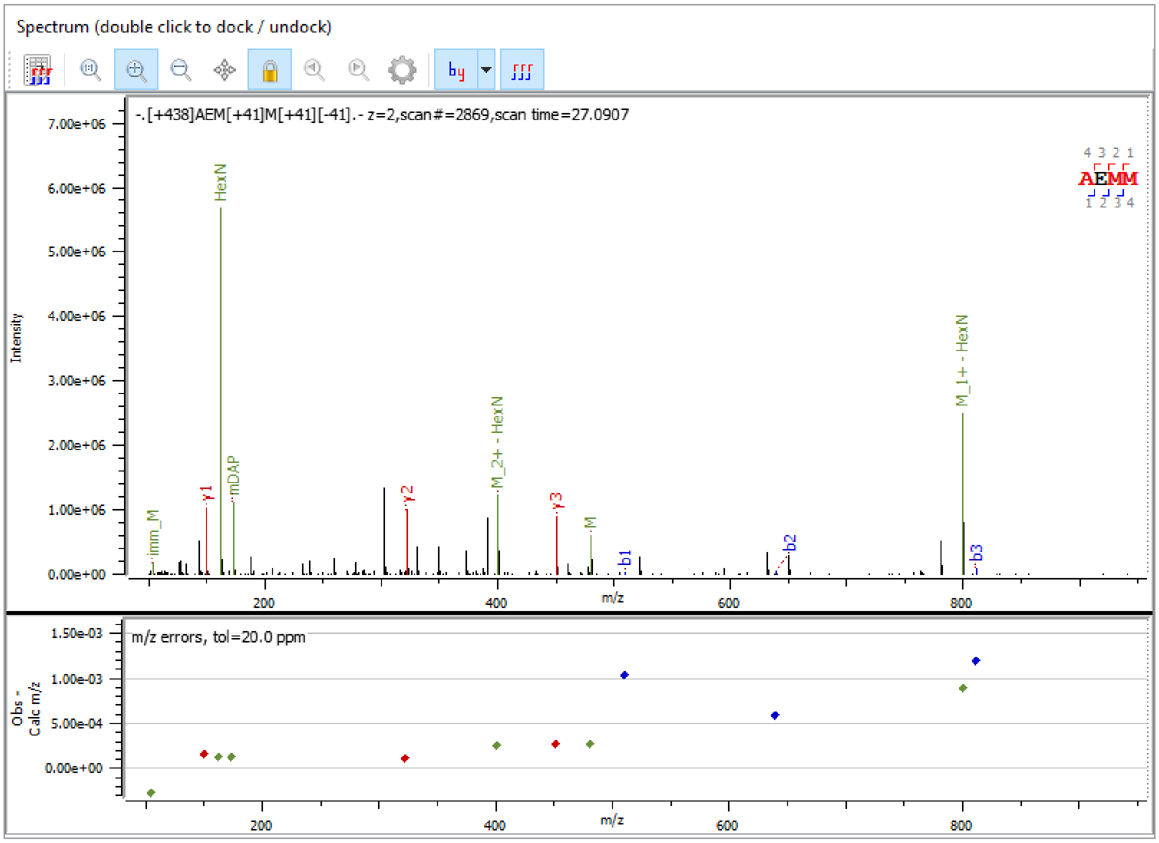
Spectrum (double click to dock / undock)
-.[+438]AEM[+41]M[+41][-41].- z=2,scan#=2869,scan time=27.0907
Intensity
m/z
HexN
mDAP
imm_M
y1
y2
y3
M_2+ - HexN
M
b1
b2
b3
M_1+ - HexN
AEMM
m/z errors, tol=20.0 ppm
Obs - Calc m/z

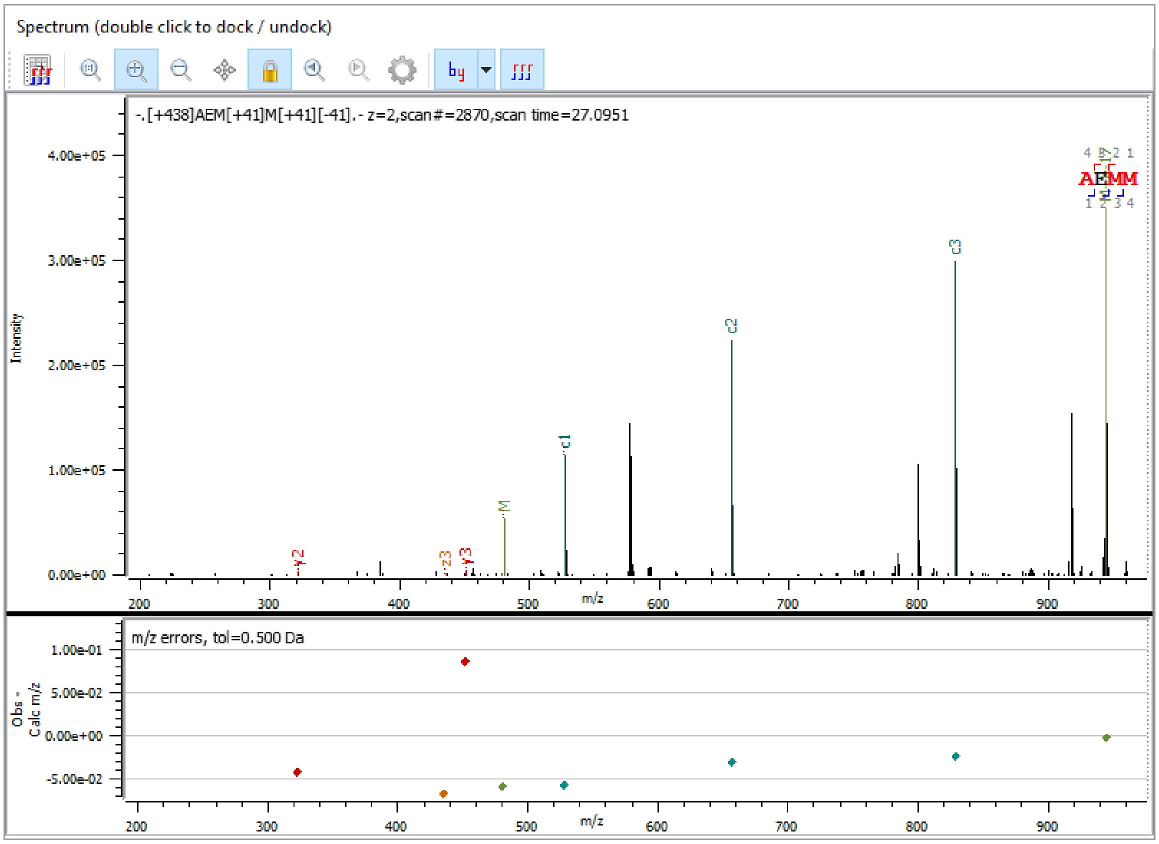
Spectrum (double click to dock / undock)
-.[+438]AEM[+41]M[+41][-41].- z=2,scan#=2870,scan time=27.0951
Intensity
m/z
y2
z3
y3
M
c1
c2
c3
AEMM
m/z errors, tol=0.500 Da
Obs - Calc m/z

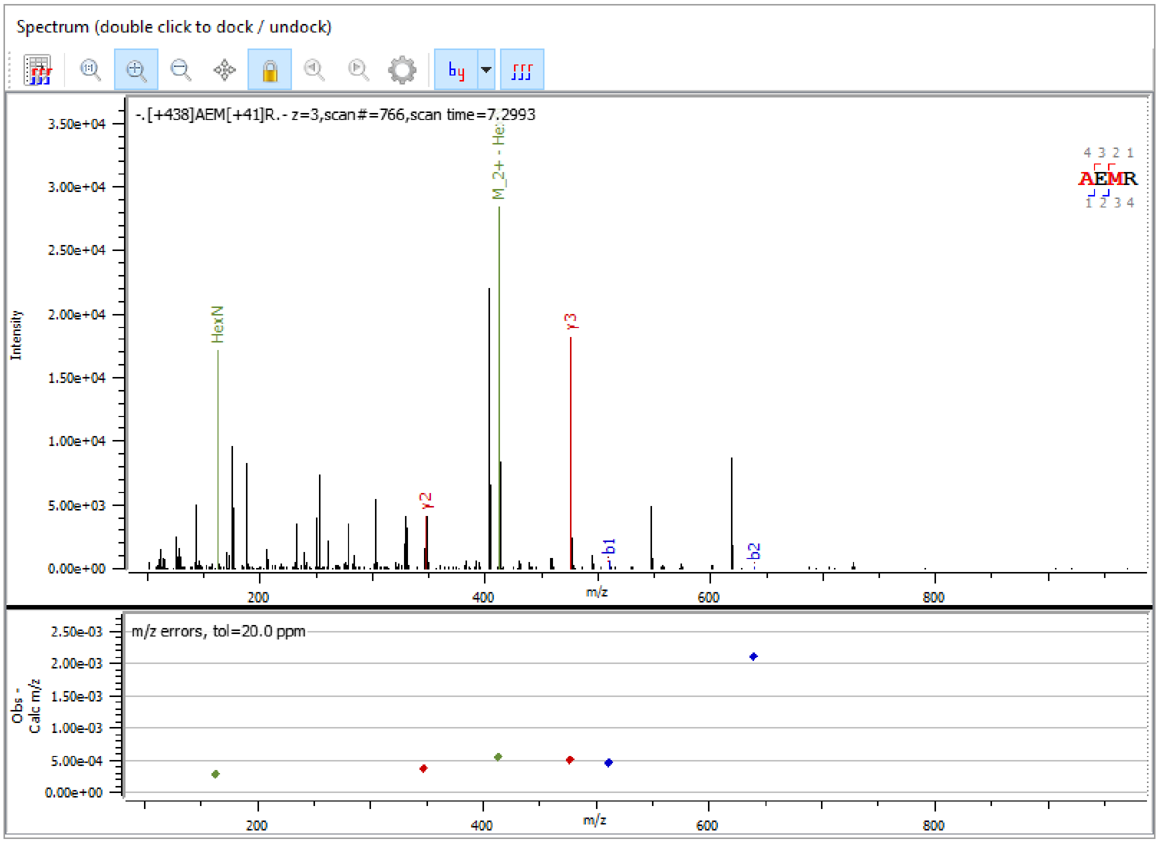
Spectrum (double click to dock / undock)
-.[+438]AEM[+41]R.- z=3,scan#=766,scan time=7.2993
HexN
M_2+ - He:
y3
y2
b1
b2
AEMR
Intensity
m/z
m/z errors, tol=20.0 ppm
Obs - Calc m/z

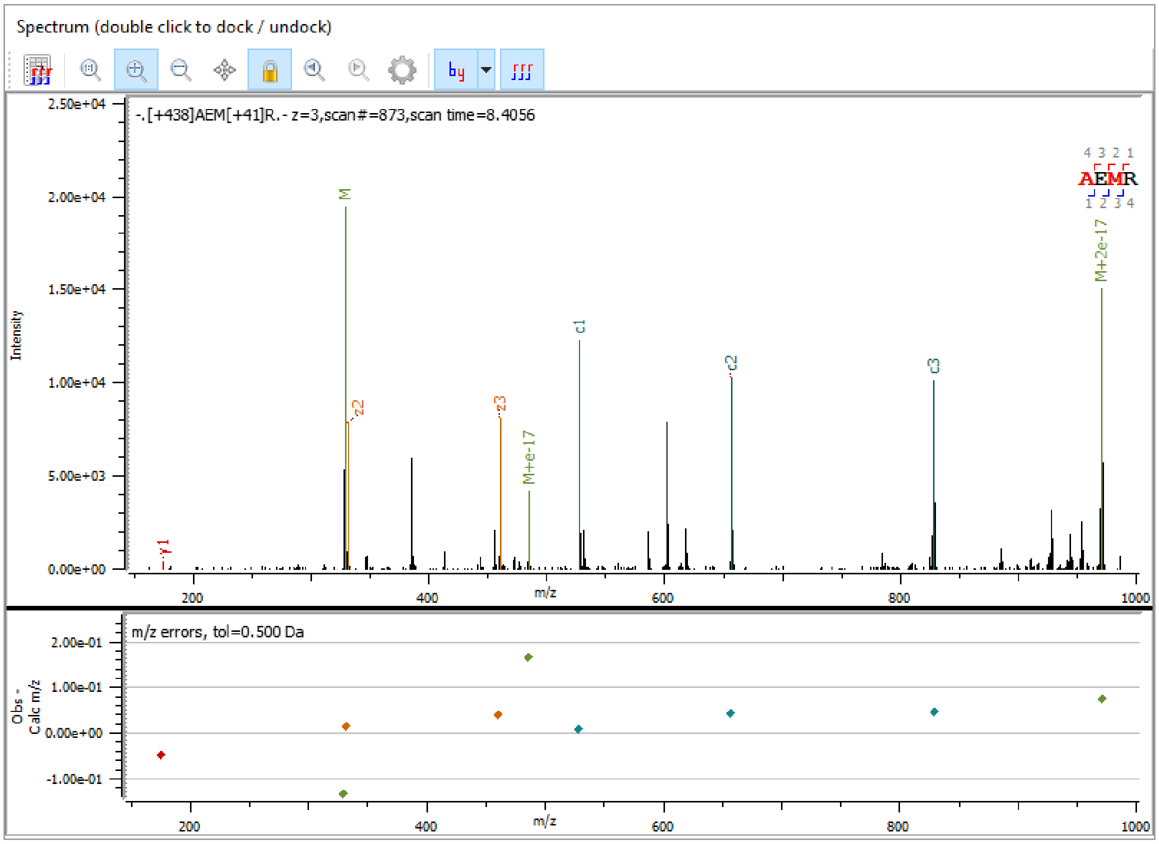
Spectrum (double click to dock / undock)
-.[+438]AEM[+41]R.- z=3,scan#=873,scan time=8.4056
M
z2
z3
M+e-17
c1
c2
c3
M+2e-17
y1
AEMR
Intensity
m/z
m/z errors, tol=0.500 Da
Obs - Calc m/z

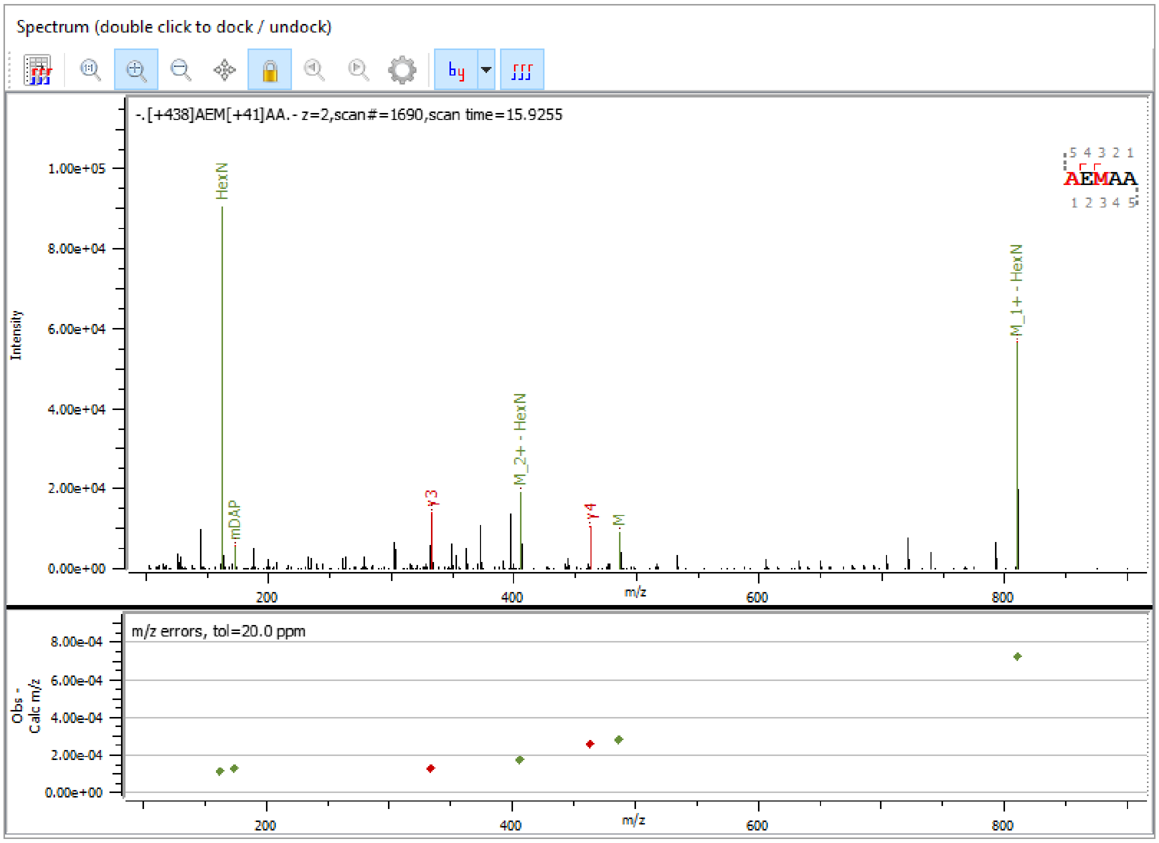
Spectrum (double click to dock / undock)
-.[+438]AEM[+41]AA.- z=2,scan#=1690,scan time=15.9255
AEMAA
HexN
mDAP
y3
M_2+ - HexN
y4
M
M_1+ - HexN
Intensity
m/z
m/z errors, tol=20.0 ppm
Obs - Calc m/z

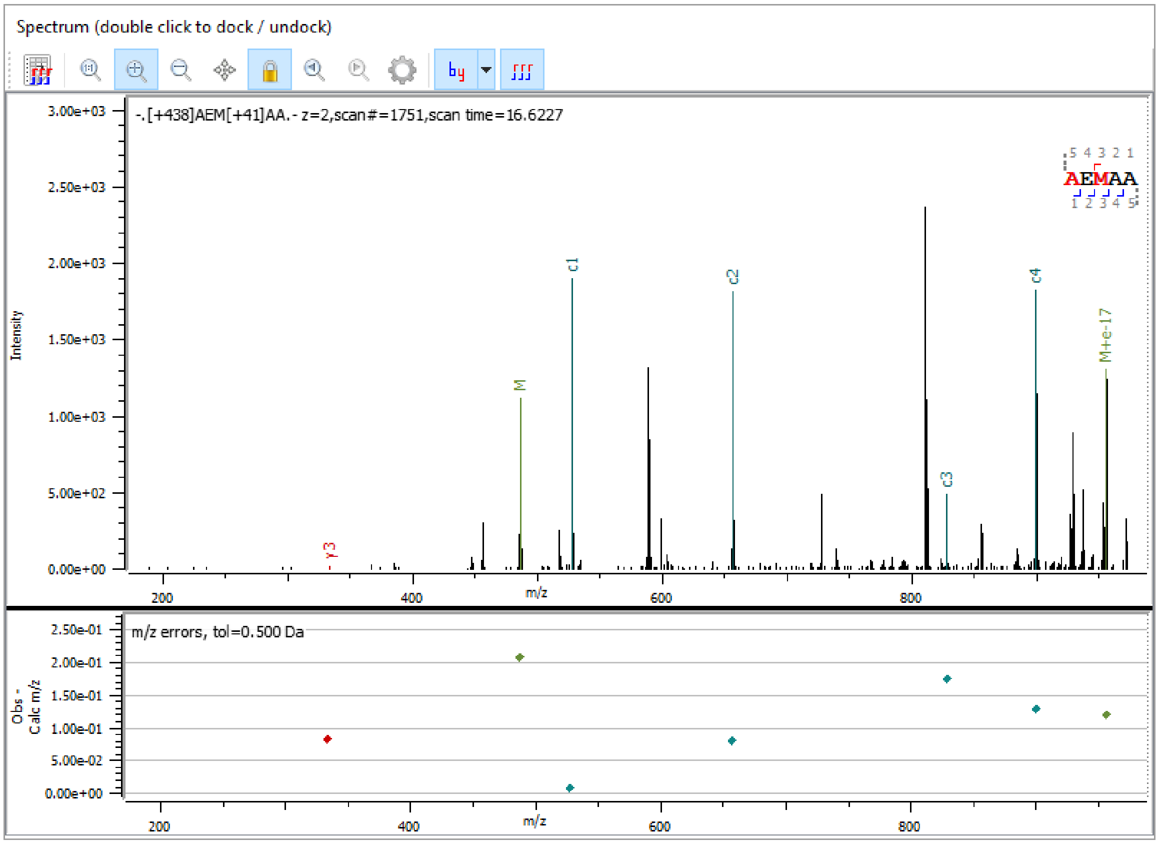
Spectrum (double click to dock / undock)
-.[+438]AEM[+41]AA.- z=2,scan#=1751,scan time=16.6227
AEMAA
y3
M
c1
c2
c3
c4
M+e-17
Intensity
m/z
m/z errors, tol=0.500 Da
Obs - Calc m/z

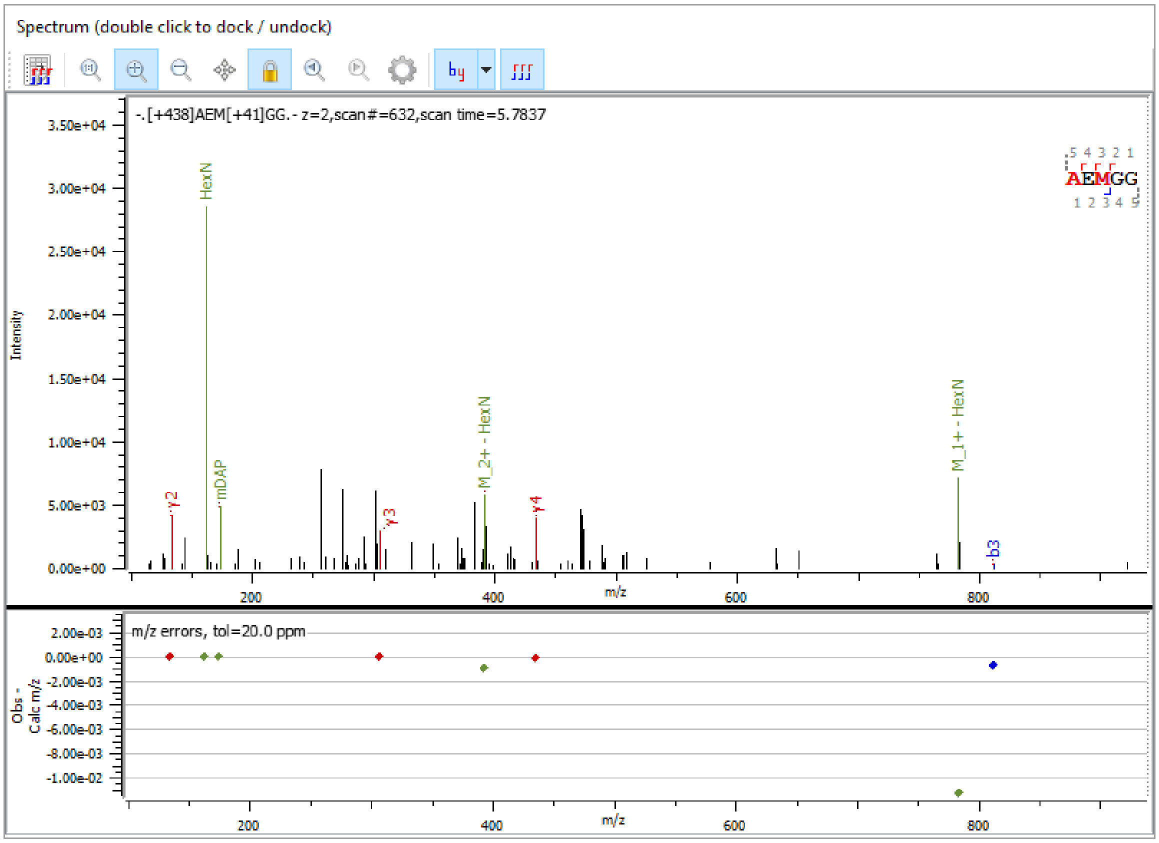
Spectrum (double click to dock / undock)
-.[+438]AEM[+41]GG.- z=2,scan#=632,scan time=5.7837
AEMGG
Intensity
HexN
mDAP
y2
y3
y4
M_2+ - HexN
M_1+ - HexN
b3
m/z
m/z errors, tol=20.0 ppm
Obs - Calc m/z

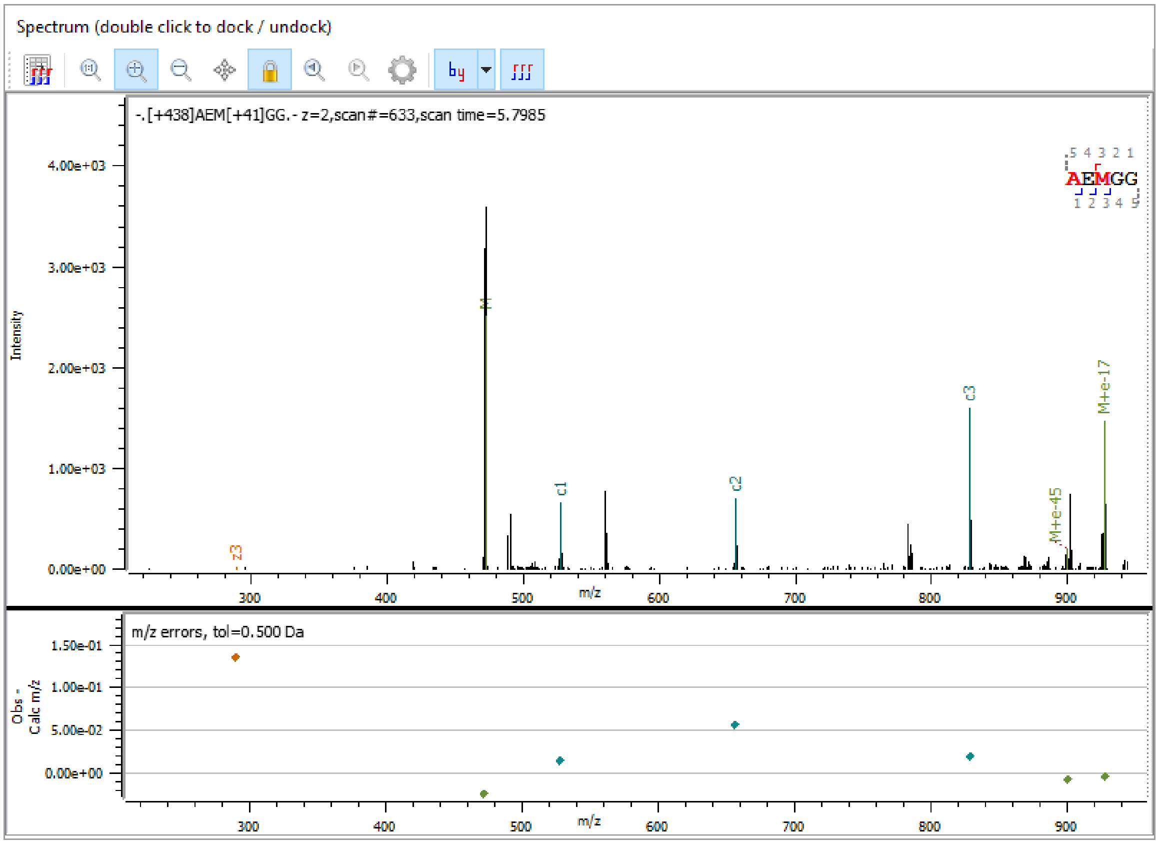
Spectrum (double click to dock / undock)
-.[+438]AEM[+41]GG.- z=2,scan#=633,scan time=5.7985
AEMGG
Intensity
z3
M
c1
c2
c3
M+e-45
M+e-17
m/z
m/z errors, tol=0.500 Da
Obs - Calc m/z

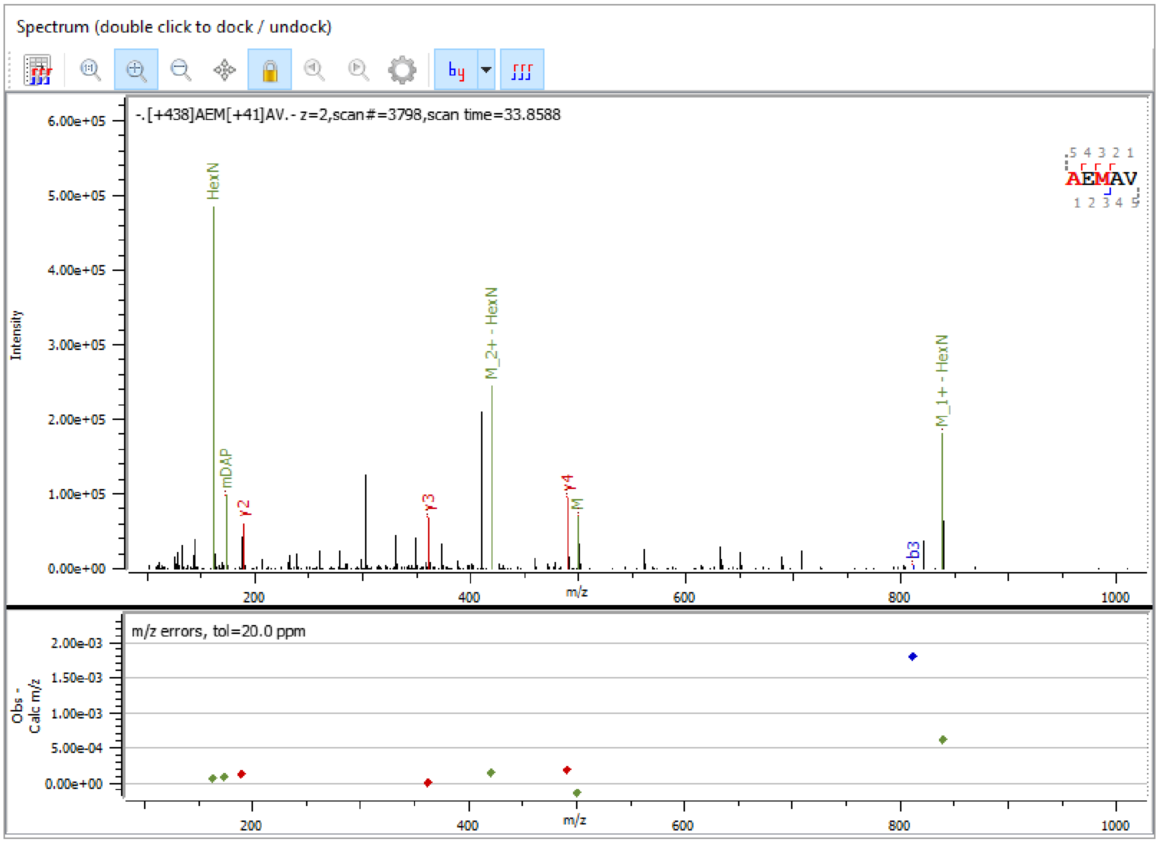
Spectrum (double click to dock / undock)
-.[+438]AEM[+41]AV.- z=2,scan#=3798,scan time=33.8588
Intensity
m/z
m/z errors, tol=20.0 ppm
Obs - Calc m/z
HexN
mDAP
y2
y3
M_2+ - HexN
y4
M
b3
M_1+ - HexN
AEMAV

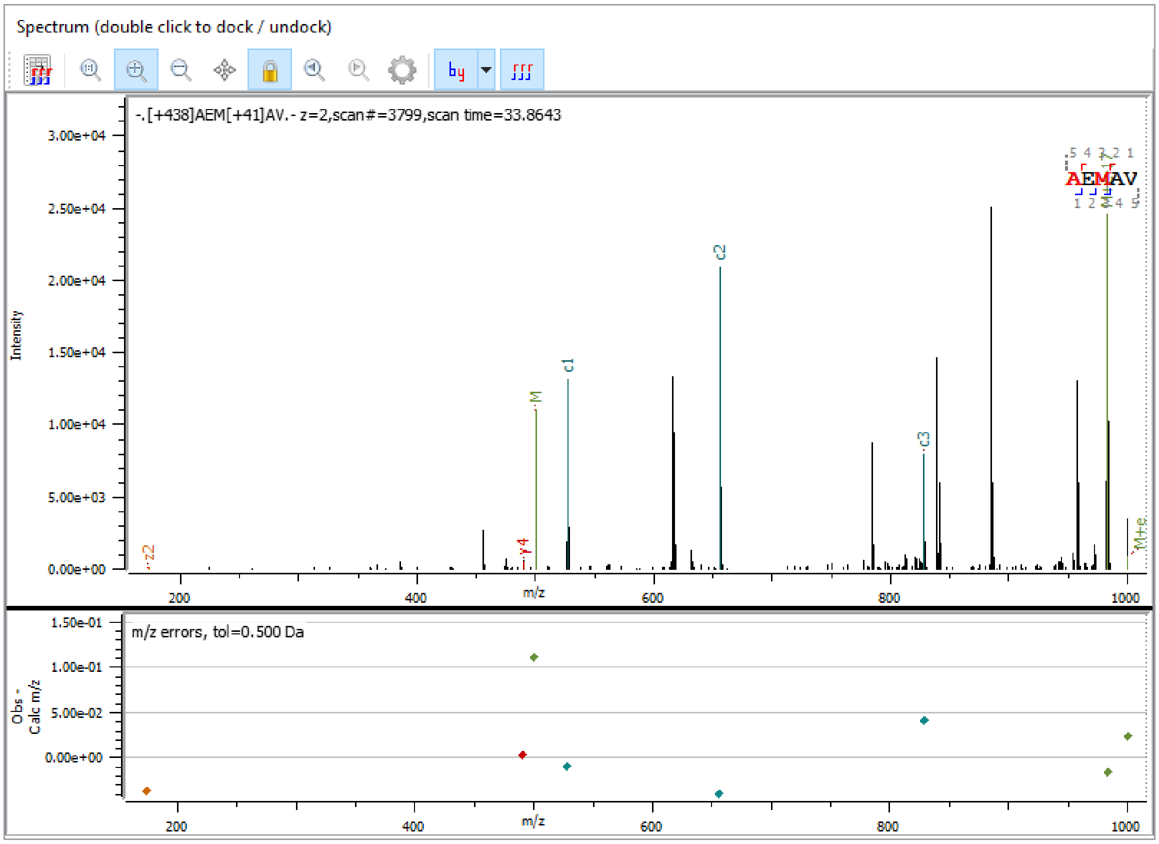
Spectrum (double click to dock / undock)
-.[+438]AEM[+41]AV.- z=2,scan#=3799,scan time=33.8643
Intensity
m/z
m/z errors, tol=0.500 Da
Obs - Calc m/z
z2
y4
M
c1
c2
c3
AEMAV

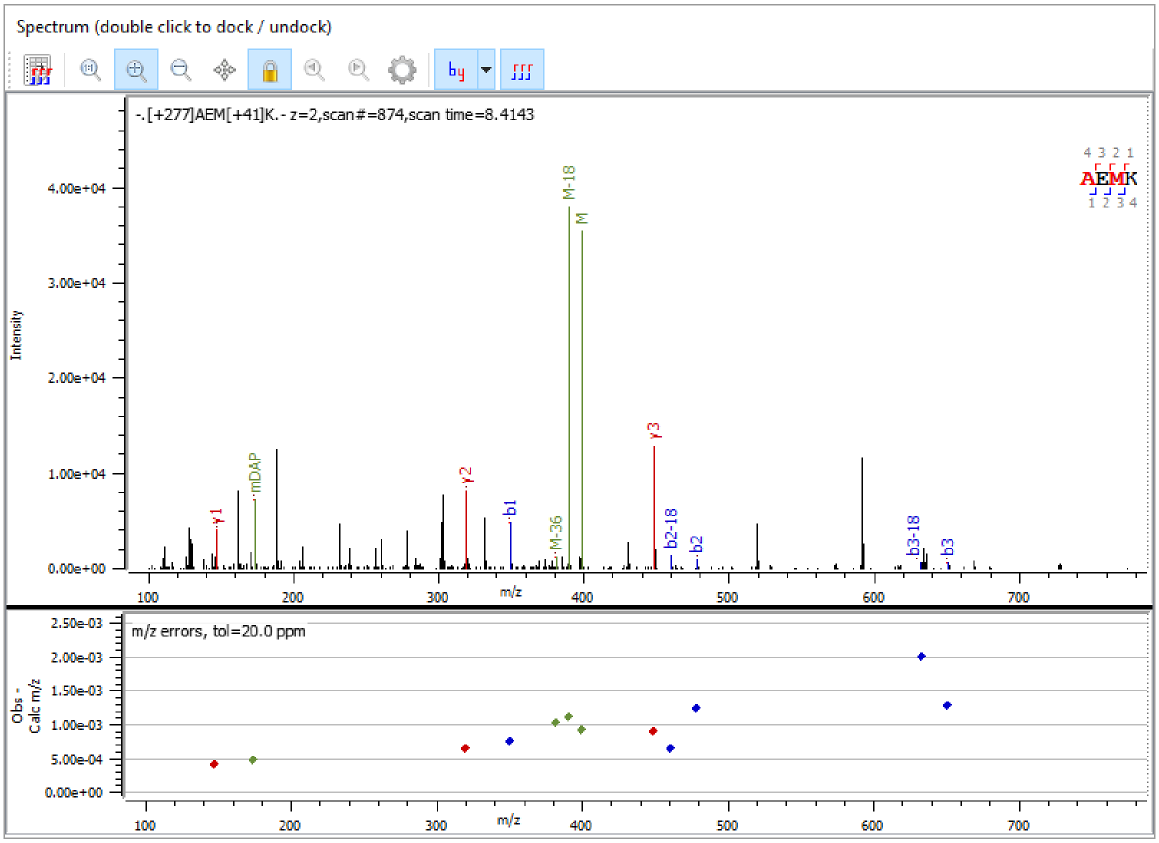
Spectrum (double click to dock / undock)
-.[+277]AEM[+41]K.- z=2,scan#=874,scan time=8.4143
Intensity
m/z
M-18
M
y3
mDAP
y2
y1
b1
M-36
b2-18
b2
b3-18
b3
m/z errors, tol=20.0 ppm
Obs - Calc m/z

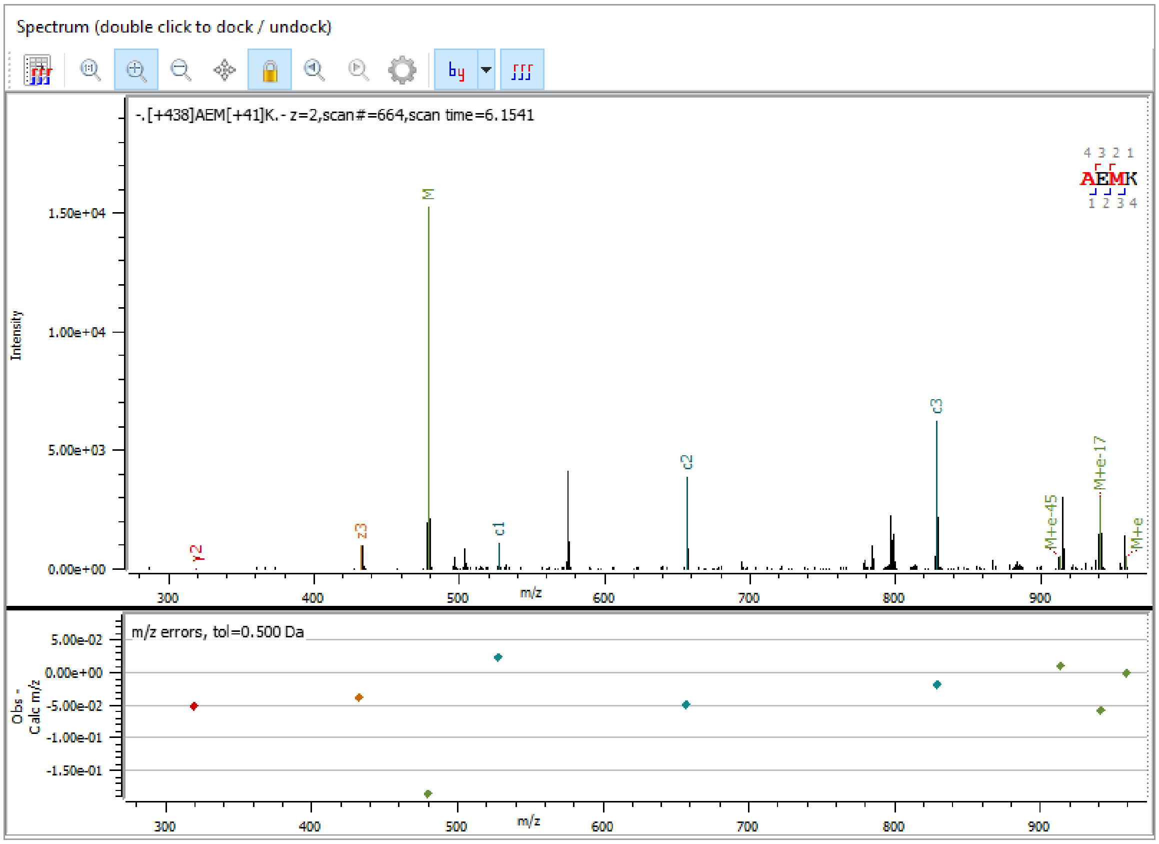
Spectrum (double click to dock / undock)
-.[+438]AEM[+41]K.- z=2,scan#=664,scan time=6.1541
Intensity
m/z
M
c3
c2
c1
z3
y2
M+e-45
M+e-17
M+e
m/z errors, tol=0.500 Da
Obs - Calc m/z

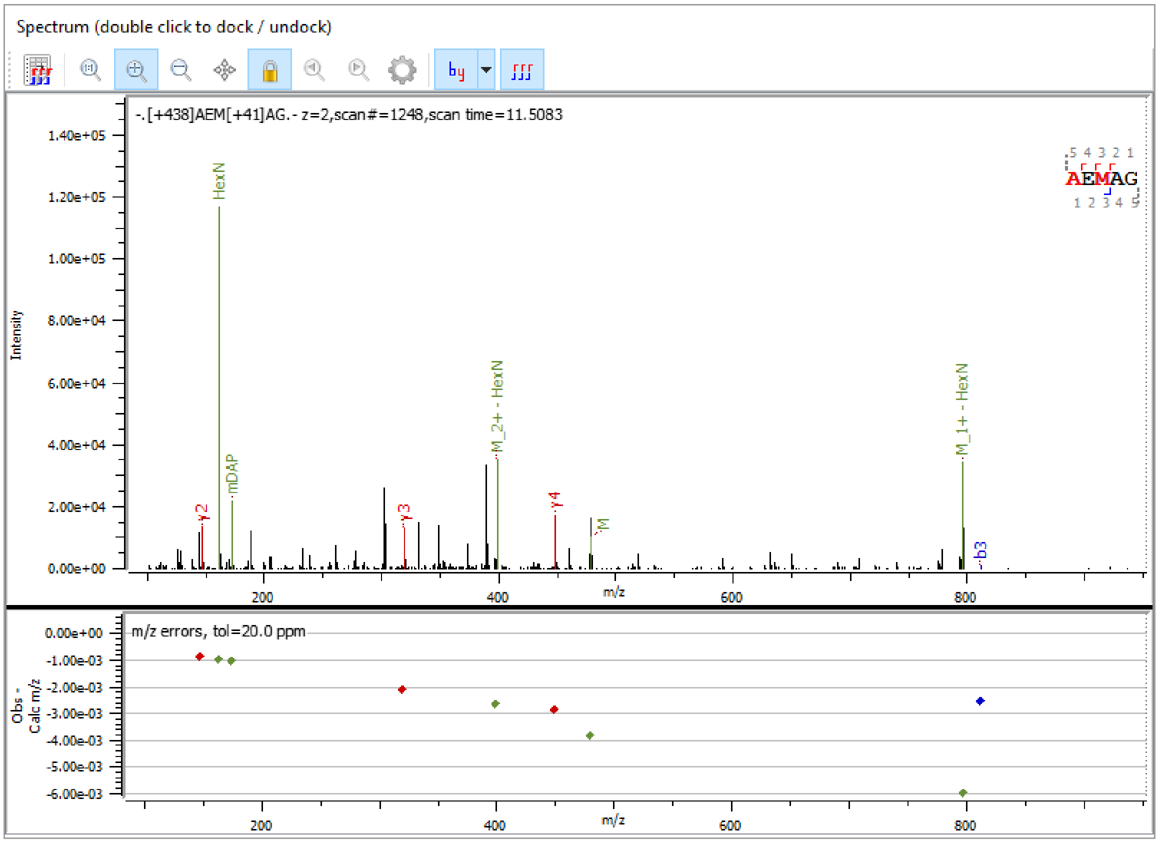
Spectrum (double click to dock / undock)
-.[+438]AEM[+41]AG.- z=2,scan#=1248,scan time=11.5083
Intensity
m/z
HexN
mDAP
M_2+ - HexN
M_1+ - HexN
y2
y3
y4
M
b3
AEMAG
m/z errors, tol=20.0 ppm
Obs - Calc m/z

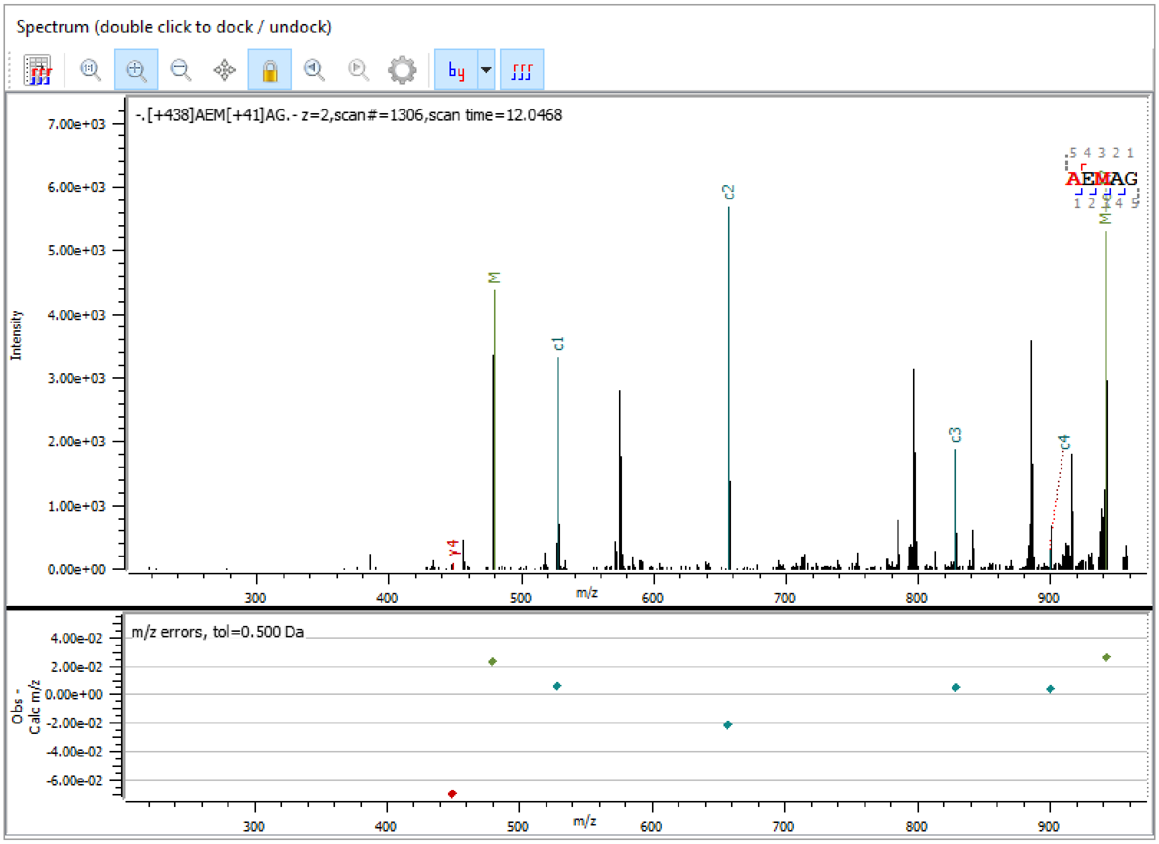
Spectrum (double click to dock / undock)
-.[+438]AEM[+41]AG.- z=2,scan#=1306,scan time=12.0468
Intensity
m/z
M
c1
c2
c3
c4
y4
AEMAG
m/z errors, tol=0.500 Da
Obs - Calc m/z

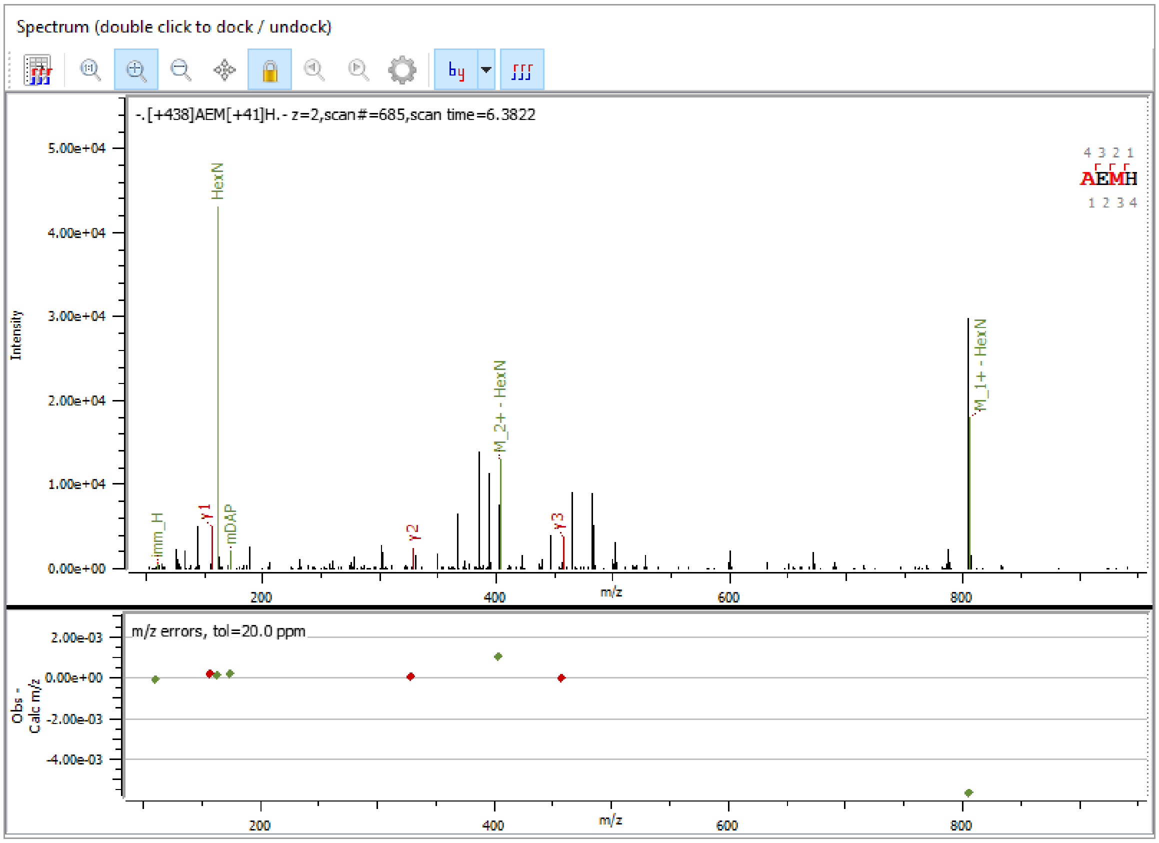
Spectrum (double click to dock / undock)
-.[+438]AEM[+41]H.- z=2,scan#=685,scan time=6.3822
Intensity
m/z
HexN
M_2+ - HexN
M_1+ - HexN
imm_H
y1
mDAP
y2
y3
AEMH
m/z errors, tol=20.0 ppm
Obs - Calc m/z

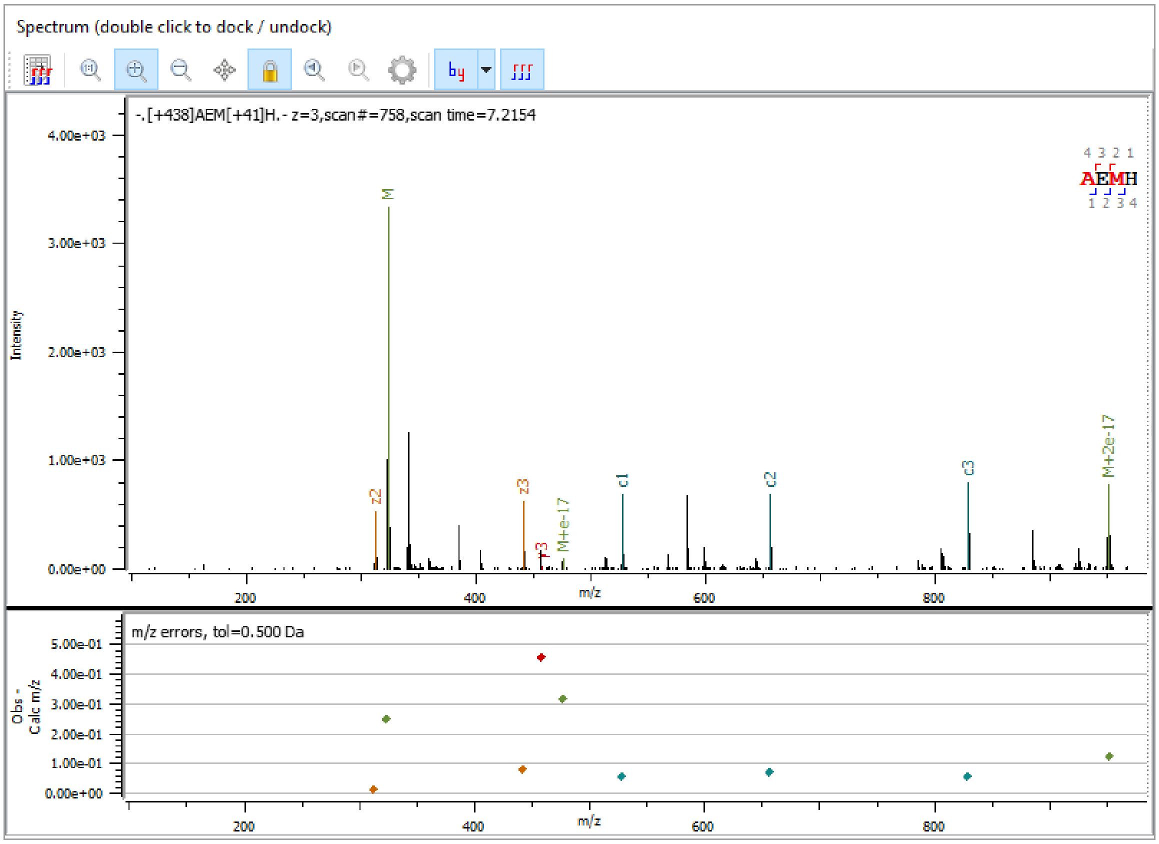
Spectrum (double click to dock / undock)
-.[+438]AEM[+41]H.- z=3,scan#=758,scan time=7.2154
Intensity
m/z
M
z2
z3
y3
M+e-17
c1
c2
c3
M+2e-17
AEMH
m/z errors, tol=0.500 Da
Obs - Calc m/z

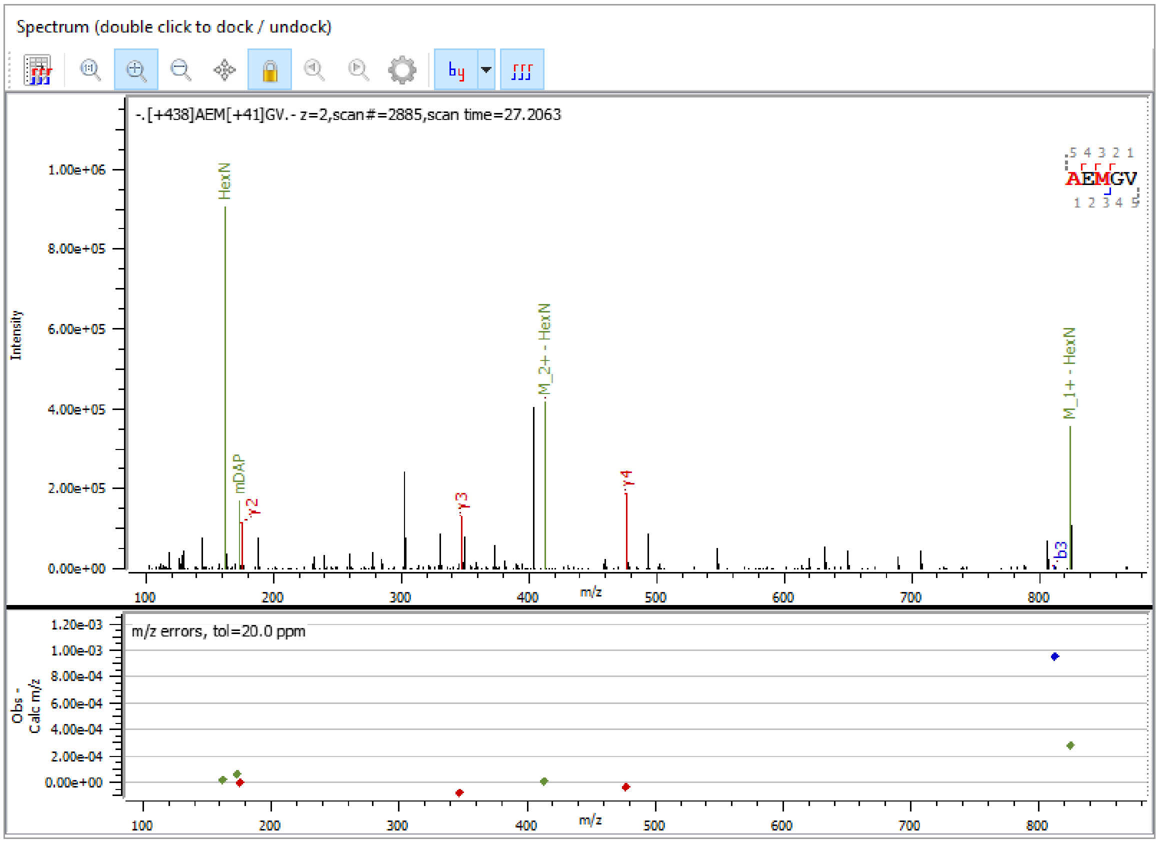
Spectrum (double click to dock / undock)
-.[+438]AEM[+41]GV.- z=2,scan#=2885,scan time=27.2063
Intensity
HexN
mDAP
y2
y3
M_2+ - HexN
y4
M_1+ - HexN
b3
AEMGV
m/z
m/z errors, tol=20.0 ppm
Obs - Calc m/z

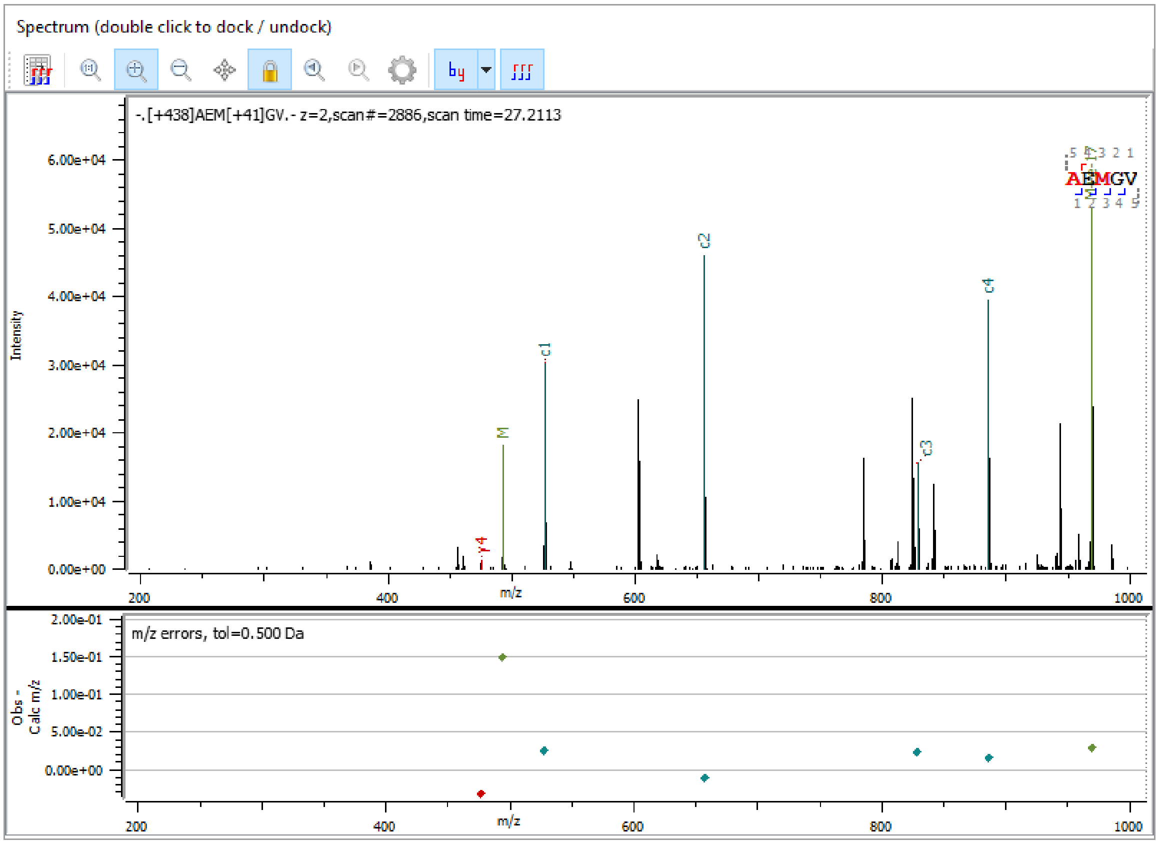
Spectrum (double click to dock / undock)
-.[+438]AEM[+41]GV.- z=2,scan#=2886,scan time=27.2113
Intensity
y4
M
c1
c2
c3
c4
AEMGV
m/z
m/z errors, tol=0.500 Da
Obs - Calc m/z

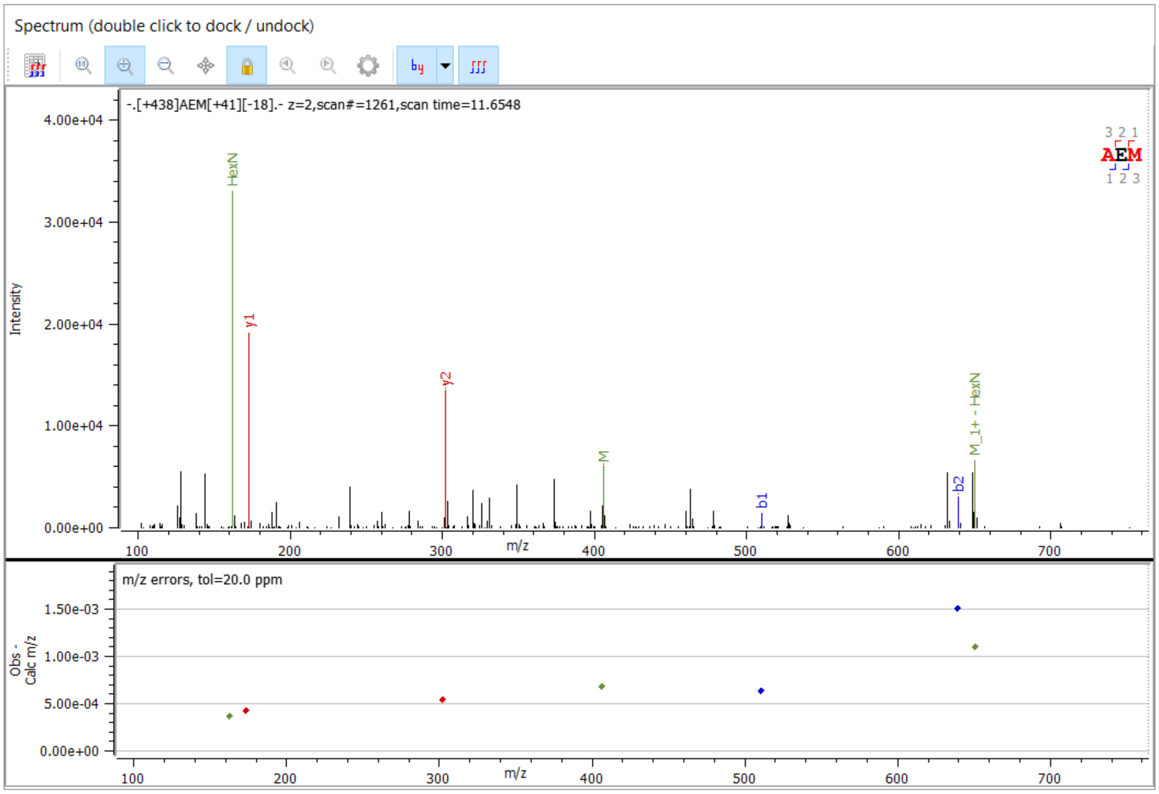

Spectrum (double click to dock / undock)
-.[+438]AEM[+41][-18].- z=2,scan#=1261,scan time=11.6548
Intensity
m/z
HexN
y1
y2
M
b1
b2
M_1+ - HexN
AEM
m/z errors, tol=20.0 ppm
Obs - Calc m/z

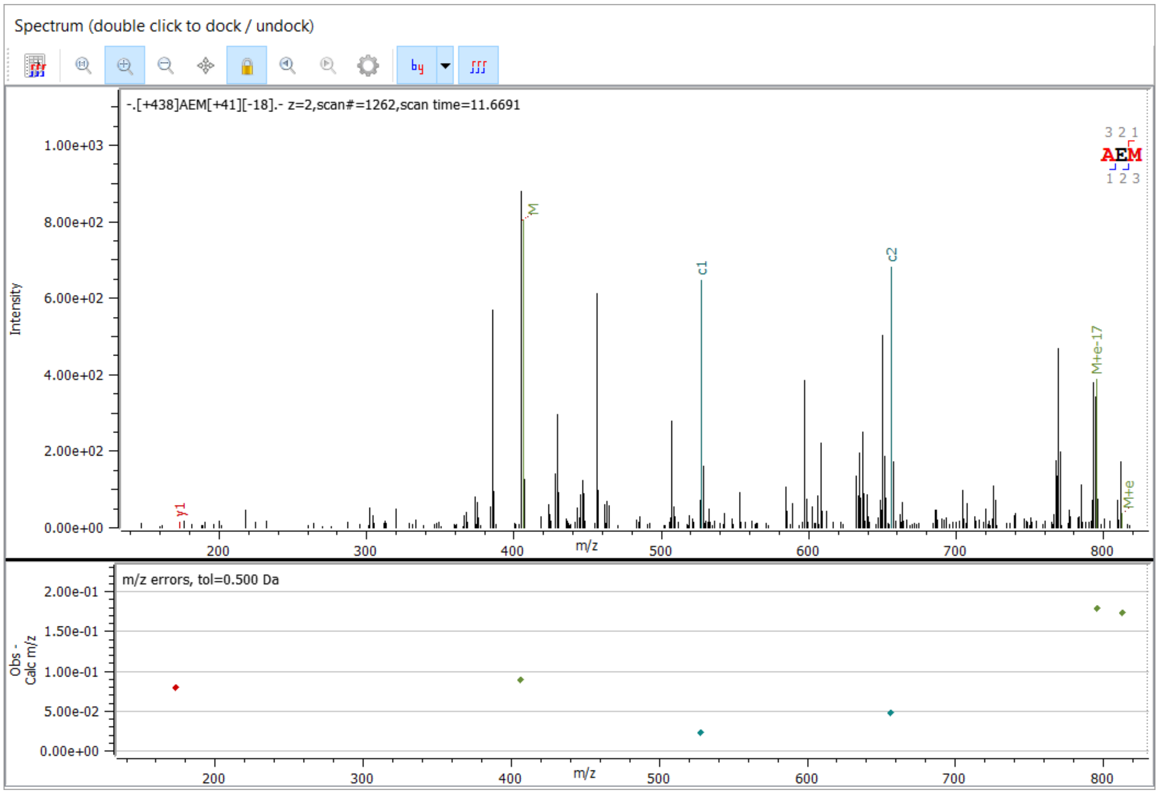

Spectrum (double click to dock / undock)
-.[+438]AEM[+41][-18].- z=2,scan#=1262,scan time=11.6691
Intensity
m/z
M
c1
c2
M+e-17
M+e
y1
AEM
m/z errors, tol=0.500 Da
Obs - Calc m/z

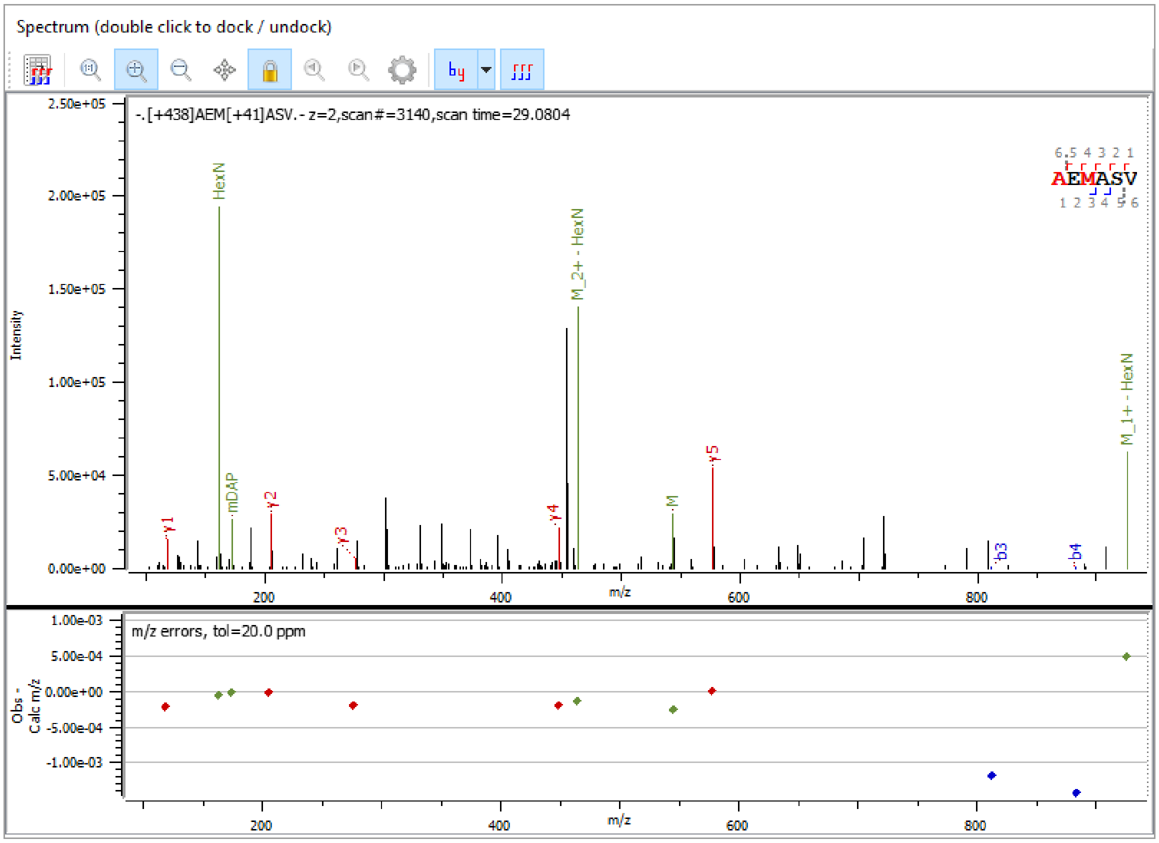
Spectrum (double click to dock / undock)
-.[+438]AEM[+41]ASV.- z=2,scan#=3140,scan time=29.0804
AEMASV
HexN
M_2+ - HexN
M_1+ - HexN
mDAP
M
y1
y2
y3
y4
y5
b3
b4
Intensity
m/z
m/z errors, tol=20.0 ppm
Obs - Calc m/z

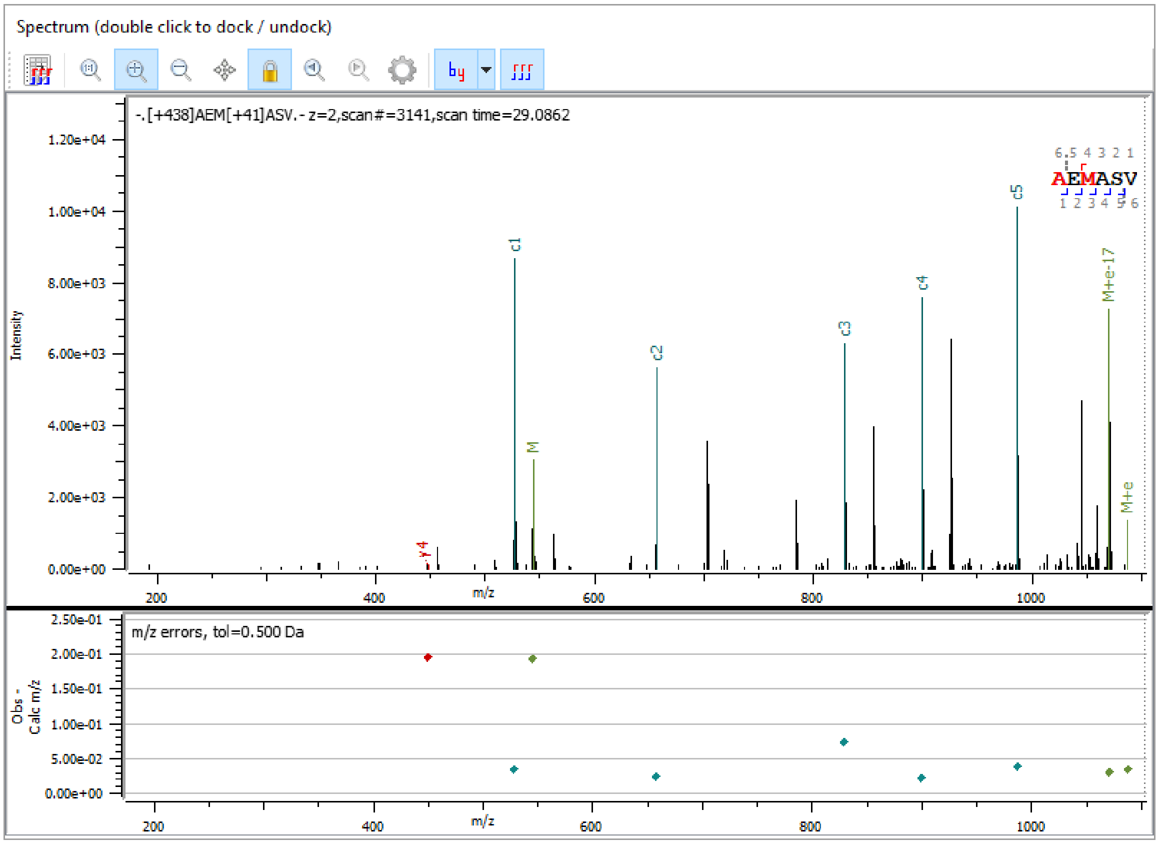
Spectrum (double click to dock / undock)
-.[+438]AEM[+41]ASV.- z=2,scan#=3141,scan time=29.0862
AEMASV
c1
c2
c3
c4
c5
M
M+e-17
M+e
y4
Intensity
m/z
m/z errors, tol=0.500 Da
Obs - Calc m/z
